# Supplementary material for: Combined translational and rotational perturbations of standing balance reveal contributions of reduced reciprocal inhibition to balance impairments in children with cerebral palsy
Source: PLoS Comput Biol. 2024 Jun 13;20(6):e1012209. doi: 10.1371/journal.pcbi.1012209 (PMC11206838; doi:10.1371/journal.pcbi.1012209)
Supplement: S1 Text — Table A. Additional information on children with cerebral palsy. GMFCS = Gross Motor Function Classification System (range 1–5) H = Hemiplegic; D = Diplegic; L = Left; R = Right; MAS = Modified Ashworth Scale for the gastrocnemii (range 1–4). S2. Full body marker set-up. Fig A. Marker set-up. S3. Muscle activity, center of mass movement, and ankle kinematics. S3.1 Exemplar trajectories. Fig B. Exemplar trajectories for center of mass movement, ankle kinematics, and muscle activity for perturbation level 2 in time bins (zones) for a child with cerebral palsy (left) and typically developing child (right) with low co-activation. FigC. Exemplar trajectories for center of mass movement, ankle kinematics, and muscle activity for perturbation level 2 in time bins (zones) for a child with cerebral palsy (left) and typically developing child (right) with high co-activation. S3.2 Muscle activity. Table B. Statistical outcome parameters (p-values) for EMG time bins for children with cerebral palsy and typically developing children. LG = lateral gastrocnemius; MG = medial gastrocnemius; SOL = soleus; TA = tibialis anterior. Significant differences (p<0.05) are indicated in bold. Table C. Post-hoc comparison for the interaction effect between group and time bin for EMG time bins for children with cerebral palsy and typically developing children. LG = Lateral Gastrocnemius; MG = Medial Gastrocnemius; SOL = Soleus; TA = Tibialis Anterior. Significant differences are indicated in bold (before Bonferroni-Holm correction). Table D. Statistical outcome parameters (p-values, Fstat, and confidence intervals) for the interaction effect between time bin and group. LG = lateral gastrocnemius; MG = medial gastrocnemius; SOL = soleus; TA = tibialis anterior; BH = Bonferroni-Holm.Significant results are indicated with Y (yes) in column six before Bonferroni-Holm correction and in column nine after Bonferroni-Holm correction. New alpha-levels defined by the Bonferroni-Holm correction are indic [file pcbi.1012209.s001.docx]

**Supporting information**

**S1. Additional information on children with cerebral palsy.**

**Table A: Additional information on children with cerebral palsy**

|  | **GMFCS** | **Hemi vs. Di** | **Tested leg** | **MAS (LG/MG)** |
| --- | --- | --- | --- | --- |
| **CP1** | 1 | D | R | 1 |
| **CP2** | 1 | H | L | 1+ |
| **CP3** | 1 | H | L | 1 |
| **CP4** | 2 | H | R | 3 |
| **CP5** | 2 | H | R | 0 |
| **CP6** | 2 | D | R | 1+ |
| **CP7** | 1 | H | L | 3 |
| **CP8** | 1 | H | R | 1 |
| **CP9** | 1 | H | R | 1 |
| **CP10** | 1 | D | R | 0 |
| **CP11** | 1 | H | L | 1+ |
| **CP12** | 1 | H | L | 1 |
| **CP13** | 1 | H | L | 0 |
| **CP14** | 2 | D | L | 1 |
| **CP15** | 1 | H | R | 1 |
| **CP16** | 1 | H | R | 1 |
| **CP17** | 1 | H | R | 1 |
| **CP18** | 1 | D | L | 1 |
| **CP19** | 2 | H | R | 1+ |
| **CP20** | 1 | D | L | 0 |
| GMFCS = Gross Motor Function Classification System (range 1-5) | | | | |
| H = Hemiplegic; D = diplegic; L = left; R = right; | | | | |
| MAS = Modified Ashworth Scale for the gastrocnemii (range 1-4) | | | | |
| **S2. Full body marker set-up.** | | | | |


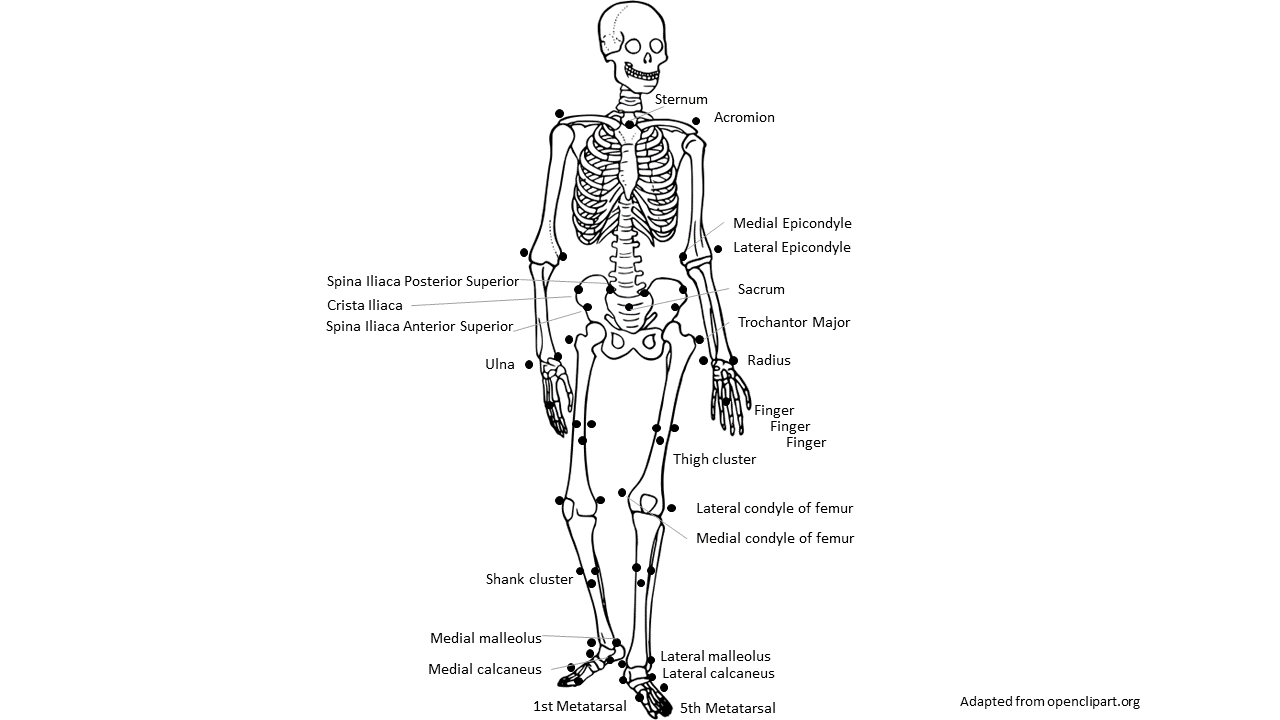


**Figure A: Marker set-up.**Figure adapted from openclipart.org.

**S3. Muscle activity, center of mass movement, and ankle kinematics**

**3.1. Exemplar trajectories**

**
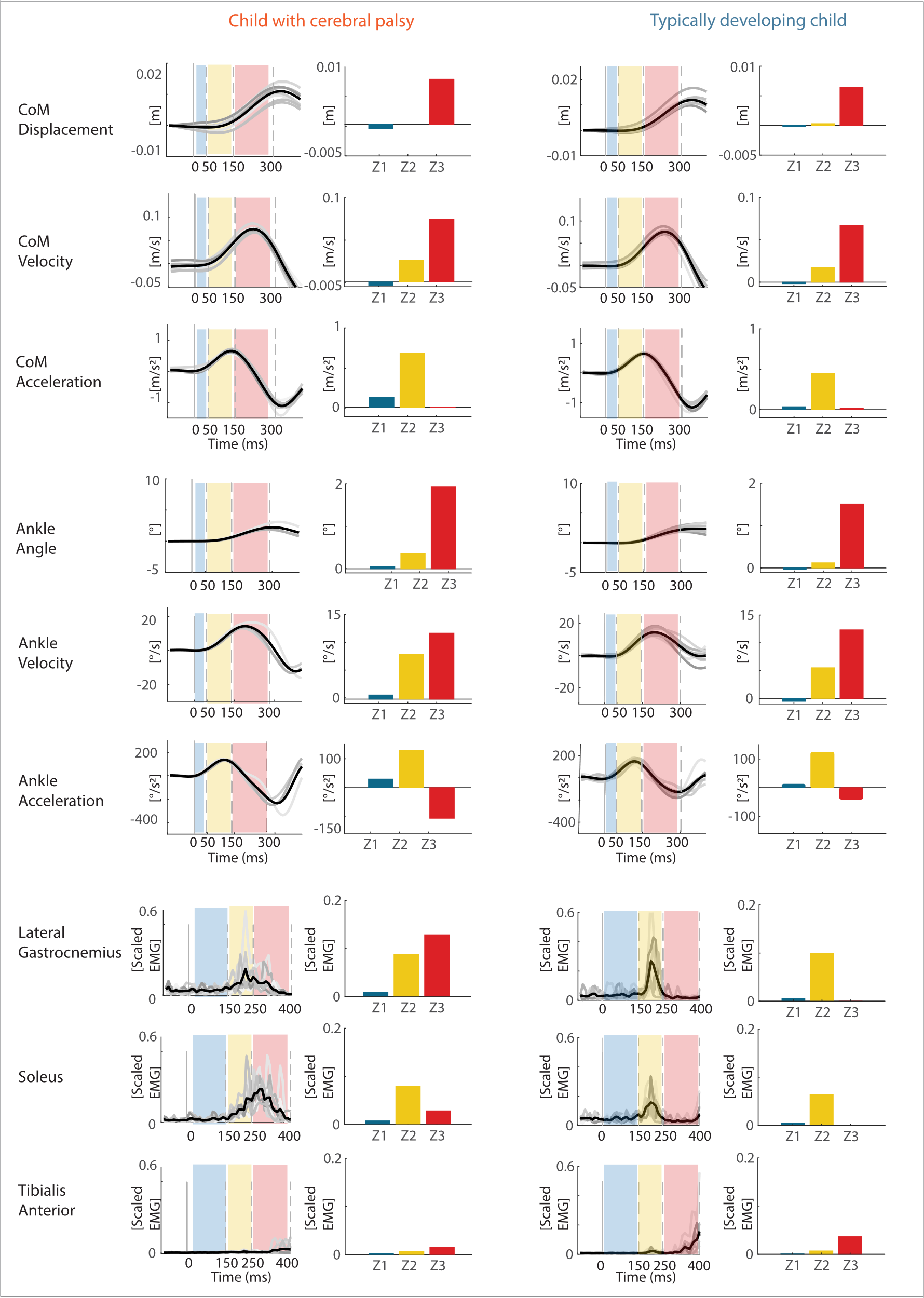
**

**Figure B: Exemplar cases for center of mass movement, ankle kinematic, and muscle activity for perturbation level 2 in time bins (zones) for a child with cerebral palsy (left) and typically developing child (right) with low co-activation.** Row 1-3: Center of mass kinematics (displacement, velocity, and acceleration) as a function of time with indication of time bins (dotted lines, colored boxes) and average trajectories (black). Time bin 1 (Z1) in blue, time bin 2 in yellow (Z2), and time bin 3 (Z3) in red; Row 4-6: Ankle angle kinematics (angle, velocity, and acceleration) as a function of time with indication of time bins and average trajectories. Row 7-9: Muscle activations as a function of time with indications of time bins and average muscle activity. Light gray traces are separate trials of one subject. The bars represent the average for each time bin for the corresponding (black) trace (average over trials) on the left.


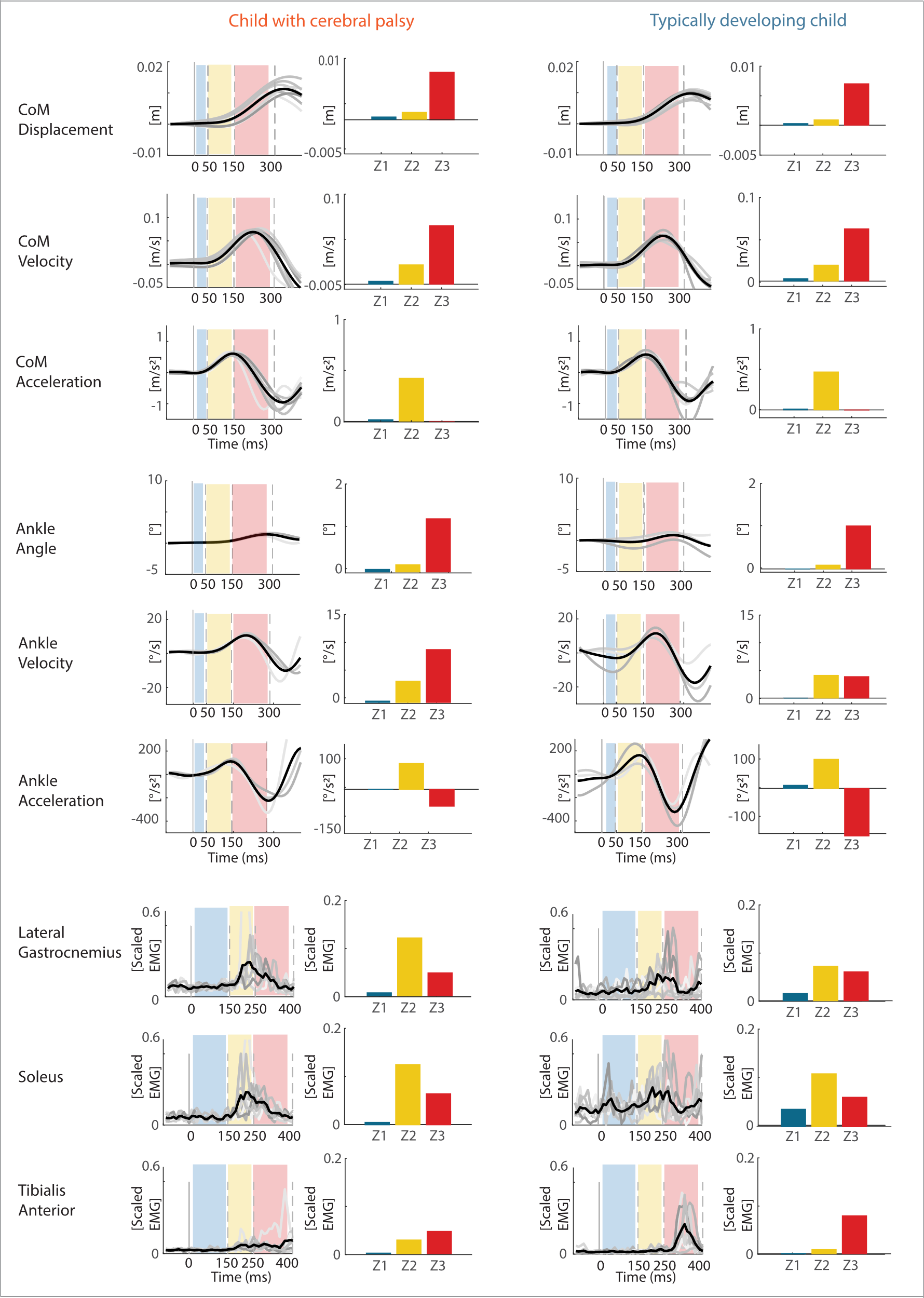


**Figure C: Exemplar trajectories for center of mass movement, ankle kinematic, and muscle activity for perturbation level 2 in time bins (zones) for a child with cerebral palsy (left) and typically developing child (right) with high co-activation.** Row 1-3: Center of mass kinematics (displacement, velocity, and acceleration) as a function of time with indication of time bins (dotted lines, colored boxes) and average trajectories (black). Time bin 1 (Z1) in blue, time bin 2 in yellow (Z2), and time bin 3 (Z3) in red; Row 4-6: Ankle angle kinematics (angle, velocity, and acceleration) as a function of time with indication of time bins and average trajectories. Row 7-9: Muscle activations as a function of time with indications of time bins and average muscle activity. Light gray traces are separate trials of one subject. The bars represent the average for each time bin for the corresponding (black) trace (average over trials) on the left.

**3.2. Muscle activity**

**Table B: Statistical outcome parameters (p-values) for EMG time bins for children with cerebral palsy and typically developing children.**

*Before Bonferroni-Holm correction:*

| **LG** | **p-value** | | | | | | | | |  | | **SOL** | |  |  | | | **p-value** | |  | |  | | |
| --- | --- | --- | --- | --- | --- | --- | --- | --- | --- | --- | --- | --- | --- | --- | --- | --- | --- | --- | --- | --- | --- | --- | --- | --- |
|  | Intercept | | | | | | Bin | Group | Bin: Group | |  | |  | | | Intercept | Bin | | Group | | Bin: Group | |  |  |
| Level 1 | 0.040 | | | | | | **6.2E-10** | 0.99 | **0.0004** | |  | | Level 1 | | | 3.70E-06 | **7.84E-13** | | 0.98 | | **0.006** | |  |  |
| Level 2 | 0.000 | | | | | | **6.8E-11** | 0.99 | **0.0002** | |  | | Level 2 | | | 8.59E-06 | **2.00E-13** | | 0.24 | | **0.006** | |  |  |
| Level 3 | 0.032 | | | | | | **2.5E-10** | 0.20 | **0.0005** | |  | | Level 3 | | | 2.32E-04 | **5.87E-13** | | 0.74 | | **0.011** | |  |  |
| Level 4 | 0.001 | | | | | | **7.3E-08** | **0.03** | **0.0358** | |  | | Level 4 | | | 6.26E-06 | **1.16E-11** | | 0.32 | | 0.069 | |  |  |
|  |  |  |  |  |  |  |  |  |  | |  | |  | | |  |  | |  | |  | |  |  |
| **MG** | **p-value** | | | | | | | | |  | | **TA** | |  | **p-value** | | | | | | | | |  |
|  | Intercept | | | | | | Bin | Group | Bin: Group | |  | |  | | | Intercept | Bin | | Group | | Bin: Group | |  |  |
| Level 1 | 3.95E-04 | | | | | | **3.61E-10** | 0.67 | 0.15 | |  | | Level 1 | | | 3.3E-01 | **1.38E-05** | | 0.62 | | **0.0057** | |  |  |
| Level 2 | 2.08E-02 | | | | | | **1.09E-12** | 0.42 | **0.00** | |  | | Level 2 | | | 4.4E-04 | **2.91E-10** | | 0.05 | | **0.0053** | |  |  |
| Level 3 | 2.94E-01 | | | | | | **3.48E-14** | 0.42 | **0.00** | |  | | Level 3 | | | 5.1E-02 | **1.57E-07** | | 0.15 | | **0.0267** | |  |  |
| Level 4 | 1.31E-03 | | | | | | **3.67E-09** | 0.83 | **0.02** | |  | | Level 4 | | | 5.9E-01 | **4.94E-09** | | 0.63 | | **0.0009** | |  |  |

LG = Lateral Gastrocnemius; MG = Medial Gastrocnemius; SOL = Soleus; TA = Tibialis Anterior.
Significant differences are indicated in bold (before Bonferroni-Holm correction).

We are mainly interested in the interaction effect between time bin and group, as this indicates differences in the muscle activity modulation between groups. Therefore, we performed a post-hoc analysis on the interaction effect between time bin and group for all muscles.

**Table C: Post-hoc comparison for the interaction effect between group and time bin for EMG time bins for children with cerebral palsy and typically developing children.**

| **LG** | **CI zone 2:Group** | |  | **LG** | **CI zone 3:Group** | |
| --- | --- | --- | --- | --- | --- | --- |
|  | LB | UB |  |  | LB | UB |
| Level 1 | -0.030 | 0.005 |  | Level 1 | **-0.041** | **-0.013** |
| Level 2 | -0.046 | 0.000 |  | Level 2 | **-0.044** | **-0.015** |
| Level 3 | -0.039 | 0.004 |  | Level 3 | **-0.038** | **-0.012** |
| Level 4 | -0.036 | 0.002 |  | Level 4 | **-0.022** | **-0.001** |
|  |  |  |  |  |  |  |
| **MG** | **CI zone 2:Group** | |  | **MG** | **CI zone 3:Group** | |
|  | LB | UB |  |  | LB | UB |
| Level 1 | -0.011 | 0.028 |  | Level 1 | -0.037 | 0.002 |
| Level 2 | -0.023 | 0.040 |  | Level 2 | **-0.052** | **-0.015** |
| Level 3 | -0.005 | 0.052 |  | Level 3 | **-0.051** | **-0.014** |
| Level 4 | -0.015 | 0.048 |  | Level 4 | **-0.037** | **-0.006** |
|  |  |  |  |  |  |  |
| **SOL** | **CI zone 2:Group** | |  | **SOL** | **CI zone 3:Group** | |
|  | LB | UB |  |  | LB | UB |
| Level 1 | -0.011 | 0.012 |  | Level 1 | **-0.050** | **-0.012** |
| Level 2 | -0.031 | 0.009 |  | Level 2 | **-0.064** | **-0.014** |
| Level 3 | -0.027 | 0.014 |  | Level 3 | **-0.055** | **-0.011** |
| Level 4 | -0.030 | 0.010 |  | Level 4 | -0.033 | -0.001 |
|  |  |  |  |  |  |  |
| **TA** | **CI zone 2:Group** | |  | **TA** | **CI zone 3:Group** | |
|  | LB | UB |  |  | LB | UB |
| Level 1 | -0.017 | 0.006 |  | Level 1 | **-0.089** | **-0.021** |
| Level 2 | -0.023 | 0.003 |  | Level 2 | **-0.098** | **-0.019** |
| Level 3 | -0.020 | 0.008 |  | Level 3 | **-0.103** | **-0.014** |
| Level 4 | -0.037 | 0.005 |  | Level 4 | **-0.120** | **-0.035** |

LG = Lateral Gastrocnemius; MG = Medial Gastrocnemius; SOL = Soleus; TA = Tibialis Anterior.
Significant differences are indicated in bold (before Bonferroni-Holm correction).

We performed the Bonferroni-Holm correction to account for simultaneous inference for the significant interaction effects (change in EMG for time bin 3).

**Table D: Statistical outcome parameters (p-values, Fstat, and confidence intervals) for the interaction effect between time bin and group.**

| **Muscle** | **Level** | **p value** | **Fstat** | **CI** | | **Significant** | **p BH** | **BH - significant** |
| --- | --- | --- | --- | --- | --- | --- | --- | --- |
|  |  |  |  | **LB** | **UB** | **(Y/N)** |  | **(Y/N)** |
| LG | L2 | **0.000** | 9.59 | -0.044 | -0.015 | **Y** | 0.0125 | **Y** |
| LG | L1 | **0.000** | 8.51 | -0.041 | -0.013 | **Y** | 0.0167 | **Y** |
| LG | L3 | **0.000** | 8.30 | -0.038 | -0.012 | **Y** | 0.0250 | **Y** |
| LG | L4 | **0.036** | 3.45 | -0.022 | -0.001 | **Y** | 0.0500 | **Y** |
|  |  |  |  |  |  |  |  |  |
| MG | L3 | **0.001** | 7.32 | -0.051 | -0.014 | **Y** | 0.0125 | **Y** |
| MG | L2 | **0.002** | 6.70 | -0.052 | -0.015 | **Y** | 0.0167 | **Y** |
| MG | L4 | **0.016** | 4.35 | -0.037 | -0.006 | **Y** | 0.0250 | **Y** |
| MG | L1 | 0.148 | 1.94 | -0.037 | 0.002 | N | 0.0500 | N |
|  |  |  |  |  |  |  |  |  |
| SOL | L2 | **0.006** | 5.41 | -0.064 | -0.014 | **Y** | 0.0125 | **Y** |
| SOL | L1 | **0.006** | 5.33 | -0.050 | -0.012 | **Y** | 0.0167 | **Y** |
| SOL | L3 | **0.011** | 4.69 | -0.055 | -0.011 | **Y** | 0.0250 | **Y** |
| SOL | L4 | 0.069 | 2.75 | -0.033 | -0.001 | N | 0.0500 | N |
|  |  |  |  |  |  |  |  |  |
| TA | L3 | **0.001** | 7.60 | -0.120 | -0.035 | **Y** | 0.0125 | **Y** |
| TA | L1 | **0.005** | 5.55 | -0.098 | -0.019 | **Y** | 0.0167 | **Y** |
| TA | L4 | **0.006** | 5.42 | -0.089 | -0.021 | **Y** | 0.0250 | **Y** |
| TA | L2 | **0.027** | 3.77 | -0.103 | -0.014 | **Y** | 0.0500 | **Y** |

LG = lateral gastrocnemius; MG = medial gastrocnemius; SOL = soleus; TA = tibialis anterior; BH = Bonferroni-Holm.Significant results are indicated with Y (yes) in column six before Bonferroni-Holm correction and in column nine after Bonferroni-Holm correction. New alpha-levels defined by the Bonferroni-Holm correction are indicated in column eight.

**3.3. Center of mass movement**

**Table E: Statistical outcome parameters (p-values) for CoM time bins for children with cerebral palsy and typically developing children.**

| **Displacement** | **p-value** | | |
| --- | --- | --- | --- |
|  | Time bin | Group | Bin: Group |
| Level 1 | **p < 0.001** | 0.60 | 0.94 |
| Level 2 | **p < 0.001** | 0.95 | 0.99 |
| Level 3 | **p < 0.001** | 0.69 | 0.97 |
| Level 4 | **p < 0.001** | 0.68 | 0.86 |
| **Velocity** | **p-value** | | |
|  | Time bin | Group | Bin: Group |
| Level 1 | **p < 0.001** | 0.21 | 0.14 |
| Level 2 | **p < 0.001** | 0.93 | 0.29 |
| Level 3 | **p < 0.001** | 0.71 | 0.18 |
| Level 4 | **p < 0.001** | 0.69 | 0.24 |
| **Acceleration** | **p-value** | | |
|  | Time bin | Group | Bin: Group |
| Level 1 | **p < 0.001** | 0.68 | 0.30 |
| Level 2 | **p < 0.001** | 0.67 | 0.13 |
| Level 3 | **p < 0.001** | 0.75 | 0.19 |
| Level 4 | **p < 0.001** | 0.78 | 0.79 |

Significant differences are indicated in bold.

We are mainly interested in the interaction effect between time bin and group, as this indicates differences in the CoM movement over time between groups. As no significant differences were found for the interaction between time bin and group, we did not perform post-hoc analysis.

**3.4. Ankle kinematics**

**Table F: Statistical outcome parameters (p-values) for ankle angle kinematics for children with cerebral palsy and typically developing children.**

| **Position** | **p-value** | | |
| --- | --- | --- | --- |
|  | Time bin | Group | Bin: Group |
| Level 1 | **p < 0.001** | 0.53 | 0.73 |
| Level 2 | **p < 0.001** | 0.95 | 0.40 |
| Level 3 | **p < 0.001** | 0.85 | 0.12 |
| Level 4 | **p < 0.001** | 0.91 | 0.46 |
| **Velocity** | **p-value** | | |
|  | Time bin | Group | Bin: Group |
| Level 1 | **p < 0.001** | 0.46 | **0.01** |
| Level 2 | **p < 0.001** | 0.77 | **p < 0.001** |
| Level 3 | **p < 0.001** | 0.81 | **0.01** |
| Level 4 | **p < 0.001** | 0.96 | 0.10 |
| **Acceleration** | **p-value** | | |
|  | Time bin | Group | Bin: Group |
| Level 1 | **p < 0.001** | 0.98 | **0.05** |
| Level 2 | **p < 0.001** | 0.42 | **0.01** |
| Level 3 | **p < 0.001** | 0.73 | 0.49 |
| Level 4 | **p < 0.001** | 0.85 | 0.21 |

Significant differences are indicated in bold.

We are mainly interested in the interaction effect between time bin and group, as this indicates differences in the change in ankle kinematics between groups. Therefore, we performed a post-hoc analysis on the interaction effect between time bin and group for angular velocity and acceleration.

**Table G: Post-hoc comparison for the interaction effect between group and time bin for angular velocity and acceleration for children with cerebral palsy and typically developing children.**

|  |  |  |  |  |  |  |
| --- | --- | --- | --- | --- | --- | --- |
| **Velocity** | **CI** | |  | **Velocity** | **CI** | |
|  | Zone2:Groep | |  |  | Zone3:Groep | |
| Level 1 | -1.47 | 1.76 |  | Level 1 | **0.60** | **3.83** |
| Level 2 | -2.57 | 2.57 |  | Level 2 | **1.46** | **6.60** |
| Level 3 | -2.28 | 4.59 |  | Level 3 | **1.53** | **8.39** |
| Level 4 | -2.94 | 4.72 |  | Level 4 | 0.13 | 7.79 |
|  |  |  |  |  |  |  |
| **Acceleration** | **CI** | |  | **Acceleration** | **CI** | |
|  | Zone2:Groep | |  |  | Zone3:Groep | |
| Level 1 | -11.66 | 24.14 |  | Level 1 | **4.01** | **39.81** |
| Level 2 | -13.21 | 51.90 |  | Level 2 | **20.46** | **85.57** |
| Level 3 | -23.60 | 59.79 |  | Level 3 | -17.69 | 65.70 |
| Level 4 | -33.41 | 56.97 |  | Level 4 | -5.63 | 86.75 |

LG = Lateral Gastrocnemius; MG = Medial Gastrocnemius; SOL = Soleus; TA = Tibialis Anterior.
Significant differences are indicated in bold (before Bonferroni-Holm correction).

We performed the Bonferroni-Holm correction to account for simultaneous inference for the significant interaction effects (change in angular velocity and acceleration for time bin 3).

**Table H: Statistical outcome parameters (p-values, Fstat, and confidence intervals) for the interaction effect between time bin 3 and group.**

|  | **Level** | **p value** | **Fstat** | **CI** | | **Significant** | **p BH** | **BH - significant** |
| --- | --- | --- | --- | --- | --- | --- | --- | --- |
|  |  |  |  | **LB** | **UB** | **(Y/N)** |  | **(Y/N)** |
| Pos | L3 | 0.119 | 2.18 | -0.02 | 0.58 | N | 0.0125 | N |
| Pos | L2 | 0.403 | 0.92 | -0.10 | 0.31 | N | 0.0167 | N |
| Pos | L4 | 0.456 | 0.79 | -0.13 | 0.51 | N | 0.0250 | N |
| Pos | L1 | 0.730 | 0.32 | -0.16 | 0.29 | N | 0.0500 | N |
|  |  |  |  |  |  |  |  |  |
| Vel | L2 | **0.002** | 6.46 | 1.46 | 6.60 | **Y** | 0.0125 | **Y** |
| Vel | L1 | **0.012** | 4.63 | 0.60 | 3.83 | **Y** | 0.0167 | **Y** |
| Vel | L3 | **0.013** | 4.50 | 1.53 | 8.39 | **Y** | 0.0250 | **Y** |
| Vel | L4 | 0.104 | 2.32 | 0.13 | 7.79 | N | 0.0500 | N |
|  |  |  |  |  |  |  |  |  |
| Acc | L2 | **0.006** | 5.35 | 20.46 | 85.57 | **Y** | 0.0125 | **Y** |
| Acc | L1 | **0.048** | 3.12 | 4.01 | 39.81 | **Y** | 0.0167 | N |
| Acc | L4 | 0.210 | 1.59 | -5.63 | 86.75 | N | 0.0250 | N |
| Acc | L3 | 0.495 | 0.71 | -17.69 | 65.70 | N | 0.0500 | N |

Pos = Ankle angle position; Vel = Ankle angle velocity; Acc = Ankle angle acceleration; BH = Bonferroni-Holm. Significant results are indicated with Y (yes) in column six before Bonferroni-Holm correction and in column nine after Bonferroni-Holm correction. New alpha-levels defined by the Bonferroni-Holm correction are indicated in column eight.

**S4. Co-contraction index**

**Table I: Statistical outcome parameters (p-values) for co-contraction index between children with cerebral palsy and typically developing children for the time frames similar as the time bins (onset-350ms after onset).**

| CCI - Short | **p-values** | | |
| --- | --- | --- | --- |
|  | Level | Group | Level:Group |
| LG - TA | **0.8825** | **0.0002** | 0.9902 |
| MG - TA | **0.9704** | **0.0034** | 0.9969 |
| SOL - TA | **0.9797** | **0.0007** | 0.9430 |

LG = lateral gastrocnemius; MG = medial gastrocnemius; SOL = soleus; TA = tibialis anterior.

Significant differences are indicated in bold.

We are mainly interested in the fixed effect of group. To account for simultaneous interference, we performed a Bonferroni-Holm correction for the effect of group.

**Table J: Statistical outcome parameters (p-values, Fstat, and confidence intervals) for the fixed effect of group.**

| **Muscle** | **Parameter** | **p value** | **Fstat** | **CI** | | **Significant** | **p BH** | **BH significant** |
| --- | --- | --- | --- | --- | --- | --- | --- | --- |
|  |  |  |  | **LB** | **UB** | **(Y/N)** |  | **(Y/N)** |
| LG-TA | Group | **0.0002** | 14.38 | -0.031 | -0.010 | **Y** | 0.0167 | **Y** |
| SOL-TA | Group | **0.0007** | 12.08 | -0.039 | -0.011 | **Y** | 0.0250 | **Y** |
| MG-TA | Group | **0.0034** | 8.88 | -0.028 | -0.006 | **Y** | 0.0500 | **Y** |

LG = lateral gastrocnemius; MG = medial gastrocnemius; SOL = soleus; TA = tibialis anterior.

BH = Bonferroni-Holm. Significant results are indicated with Y (yes) in column six before Bonferroni-Holm correction and in column nine after Bonferroni-Holm correction. New alpha-levels defined by the Bonferroni-Holm correction are indicated in column eight.

**Table K: Statistical outcome parameters (p-values) for co-contraction index between children with cerebral palsy and typically developing children for the time frames similar as the sensorimotor response model.**

| CCI - long | **p-values** | | |
| --- | --- | --- | --- |
|  | Level | Group | Level:Group |
| LG - TA | 0.14 | **p < 0.001** | 0.66 |
| MG - TA | **0.02** | **p < 0.001** | 0.11 |
| SOL - TA | 0.10 | **0.001** | 0.53 |

LG = lateral gastrocnemius; MG = medial gastrocnemius; SOL = soleus; TA = tibialis anterior.

Significant differences are indicated in bold.

We are mainly interested in the fixed effect of group. To account for simultaneous interference, we performed a Bonferroni-Holm correction for the effect of group.

**Table L: Statistical outcome parameters (p-values, Fstat, and confidence intervals) for the fixed effect of group.**

| **Muscle** | **p-value** | **Fstat** | **CI** | | **Significant** | **p BH** | **BH - significant** | |
| --- | --- | --- | --- | --- | --- | --- | --- | --- |
|  |  |  | **LB** | **UB** | **Y/N** |  | **Y/N** |  |
| MG-TA | **0.0000** | 23.71 | -0.021 | -0.009 | **Y** | 0.0167 | **Y** |  |
| SOL-TA | **0.0000** | 21.21 | -0.219 | -0.116 | **Y** | 0.0250 | **Y** |  |
| LG-TA | **0.0004** | 13.19 | -0.025 | -0.007 | **Y** | 0.0500 | **Y** |  |

LG = lateral gastrocnemius; MG = medial gastrocnemius; SOL = soleus; TA = tibialis anterior.

BH = Bonferroni-Holm. Significant results are indicated with Y (yes) in column six before Bonferroni-Holm correction and in column nine after Bonferroni-Holm correction. New alpha-levels defined by the Bonferroni-Holm correction are indicated in column eight.


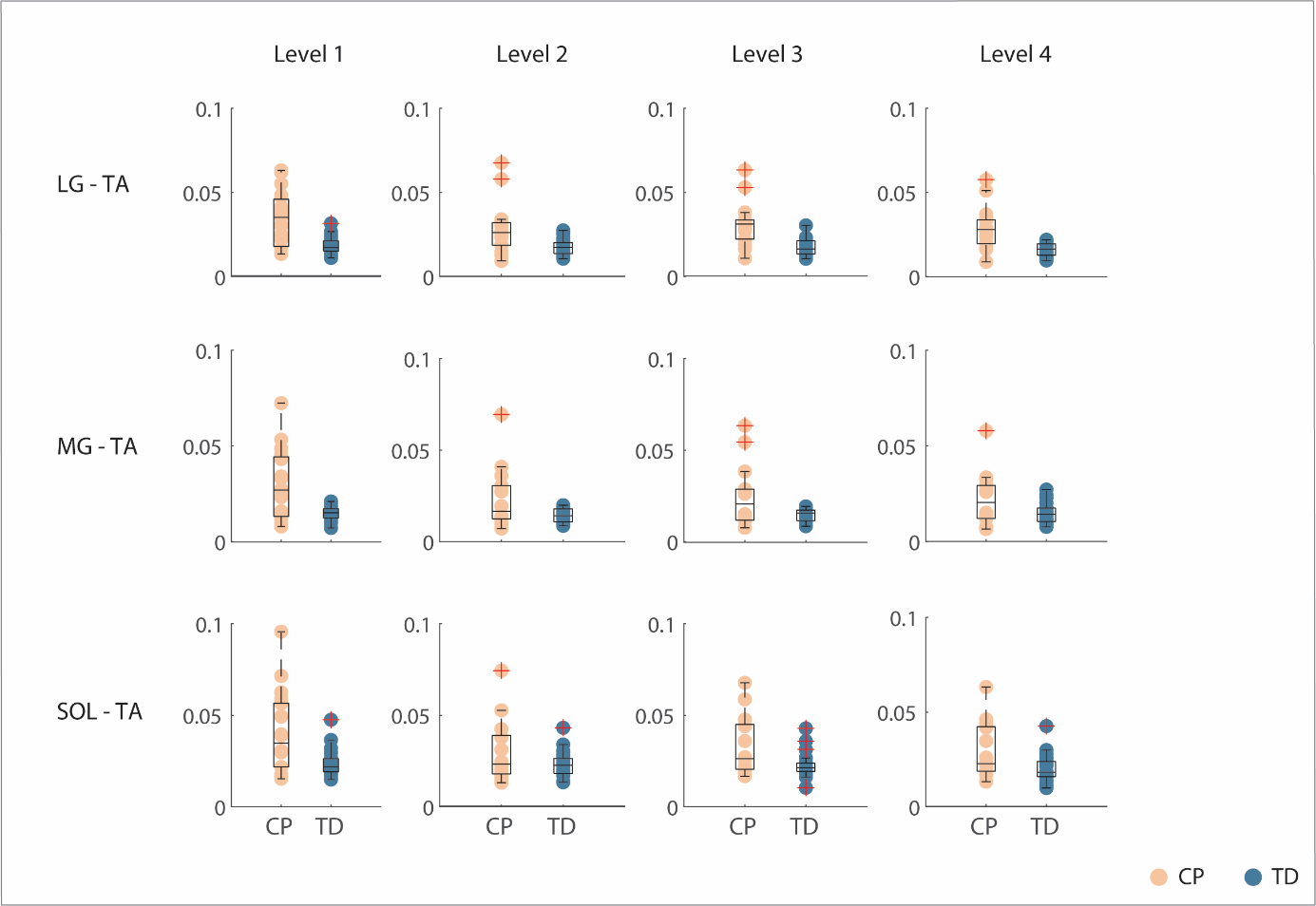


**Figure D: Co-contraction index for 0.5s before perturbation onset until 1.5s after perturbation onset.** Children with cerebral palsy (CP) in orange, typically developing (TD) children in blue. Boxplots in black indicate median and interquartile range and dots represent individual scores. Groups are significantly different across all levels for all muscle pairs.

LG = lateral gastrocnemius; MG = medial gastrocnemius; SOL = soleus; TA = tibialis anterior.

**S5. Sensorimotor response model**

**5.1 Exemplar responses**

**
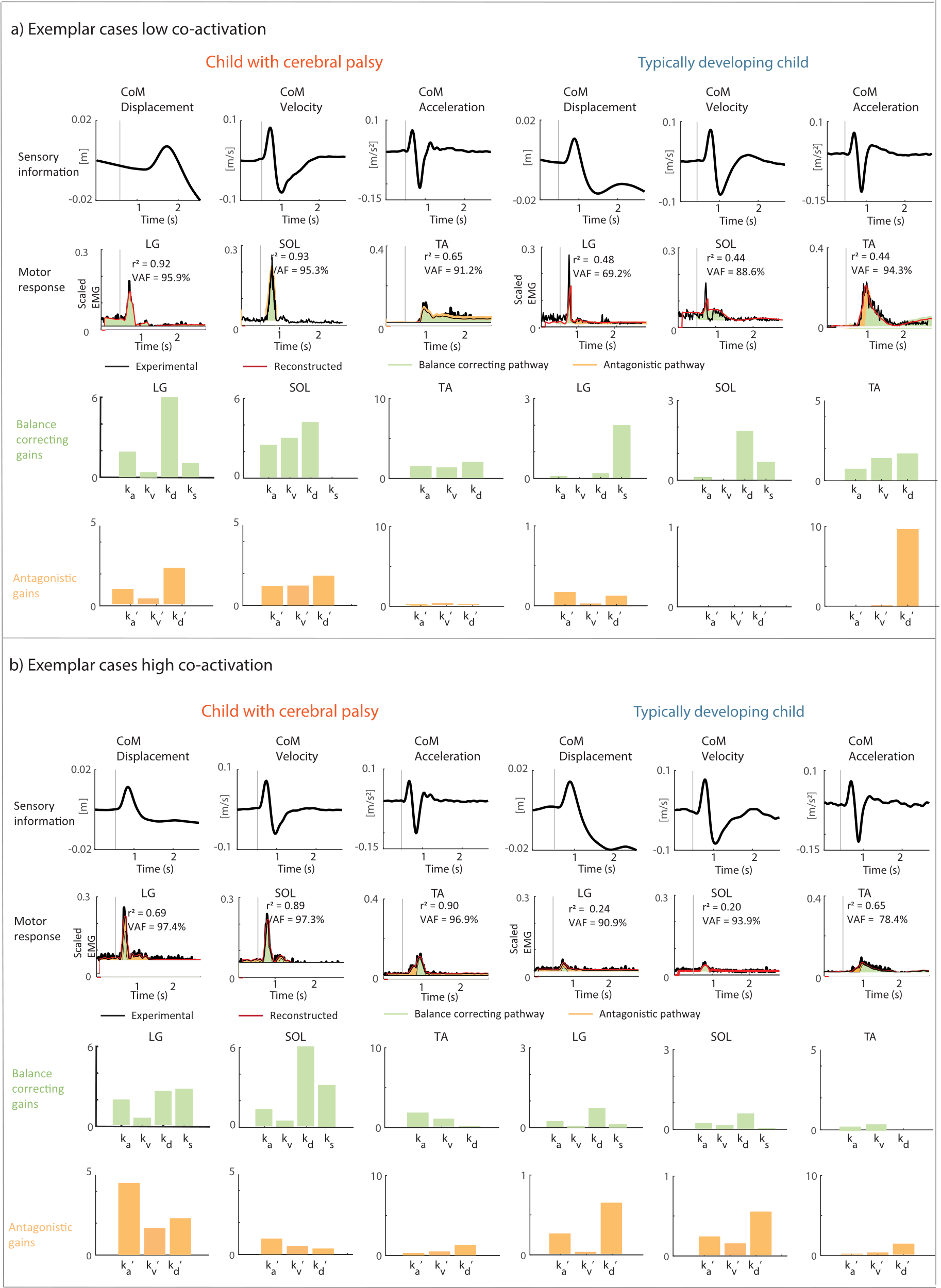
**

**Figure E: Exemplar trajectories for the sensorimotor response model for children with low co-activation (a) and children with high co-activation (b).** Exemplar cases with the extended sensorimotor response model for two children with cerebral palsy (left) and two typically developing children (right) for perturbation level 2. Top row: center of mass kinematics; second row: measured (black) and reconstructed (red) muscle activity signals with balance correcting contribution in green and antagonistic contribution in orange; third row: balance correcting gains; bottom row: antagonistic gains (i.e., prime gains). Grey line indicated onset of perturbation. LG = lateral gastrocnemius; SOL = soleus; TA = tibialis anterior.

**5.2. Goodness of fit values**

Goodness of fit values are high for both children with cerebral palsy and typically developing children.

**Table M: Goodness of fit and error scores (mean and standard deviations) for the extended sensorimotor response model for children with cerebral palsy and typically developing children.**

| **Goodness of fit** | **CP** | | **TD** | |
| --- | --- | --- | --- | --- |
|  | mean | (± SD) | mean | (± SD) |
|  | R^2^ | | | |
| LG | 0.53 | 0.20 | 0.43 | 0.20 |
| MG | 0.62 | 0.20 | 0.57 | 0.18 |
| SOL | 0.59 | 0.22 | 0.44 | 0.21 |
| TA | 0.67 | 0.20 | 0.56 | 0.21 |
|  | VAF [%] | | | |
| LG | 89.52 | 8.23 | 89.91 | 6.61 |
| MG | 88.86 | 8.21 | 80.39 | 10.39 |
| SOL | 92.44 | 4.44 | 90.70 | 4.14 |
| TA | 89.33 | 6.63 | 86.47 | 7.14 |
|  | RMSE | | | |
| LG | 0.016 | 0.009 | 0.010 | 0.006 |
| MG | 0.015 | 0.010 | 0.019 | 0.015 |
| SOL | 0.016 | 0.008 | 0.016 | 0.007 |
| TA | 0.023 | 0.015 | 0.015 | 0.010 |

R^2^: R-squared, indicating fit with overall pattern; VAF= variance accounted for, indicating fit with amplitude of response activity; RMSE = root mean square error, indicating absolute error between measured and reconstructed signal

LG = lateral gastrocnemius; MG = medial gastrocnemius; SOL = soleus; TA = tibialis anterior

CP = cerebral palsy; TD = typically developing

**5.3. Improvement in fit when adding antagonistic muscle pathways**

**Table N: Improvement in fit (mean and standard deviations) when adding antagonistic feedback pathways (extended model vs. simple model) for children with cerebral palsy and typically developing children.**

| **Fit improvement [%]** | **CP** | | **TD** | | **p-value** | | |
| --- | --- | --- | --- | --- | --- | --- | --- |
|  | Mean | (± SD) | Mean | (± SD) | Level | Group | Level:Group |
| **LG** | 26.4 | 3.7 | 8.2 | 4.5 | 0.26 | 0.27 | 0.20 |
| **MG** | 23.4 | 4.5 | 5.5 | 2.6 | 0.31 | 0.06 | 0.33 |
| **SOL** | 26.0 | 8.5 | 12.1 | 3.8 | 0.49 | **0.01** | 0.75 |
| **TA** | 63.2 | 6.8 | 43.9 | 5.1 | **0.02** | **0.04** | 0.36 |

LG = lateral gastrocnemius; MG = medial gastrocnemius; SOL = soleus; TA = tibialis anterior

CP = cerebral palsy; TD = typically developing

Significant differences (p<0.05) are indicated in bold.

**5.4. Feedback gains**

**Table O: Statistical outcome parameters (p-values) for feedback gains for the extended model for children with cerebral palsy and typically developing children.**

| **LG** | **p-value** | | |  | **TA** | **p-value** | | |
| --- | --- | --- | --- | --- | --- | --- | --- | --- |
|  | Level | Group | Level:Group |  |  | Level | Group | Level:Group |
| k_a_ | **0.000** | 0.543 | 0.406 |  | k_a_ | **0.002** | 0.099 | **0.032** |
| k_v_ | **0.002** | **0.004** | **0.020** |  | k_v_ | **0.003** | **0.002** | **0.047** |
| k_p_ | **0.001** | **0.018** | 0.097 |  | k_p_ | 0.093 | 0.259 | 0.099 |
| k'_a_ | **0.046** | 0.226 | 0.555 |  | k'_a_ | 0.673 | 0.938 | 0.489 |
| k'_v_ | 0.050 | **0.003** | 0.189 |  | k'_v_ | 0.065 | **0.001** | 0.439 |
| k'_p_ | 0.364 | 0.129 | 0.585 |  | k'_p_ | 0.303 | **0.036** | 0.809 |
| k_s_ | **0.007** | 0.437 | 0.533 |  |  |  |  |  |
|  |  |  |  |  |  |  |  |  |
| **MG** | **p-value** | | |  | **SOL** | **p-value** | | |
|  | Level | Group | Level:Group |  |  | Level | Group | Level:Group |
| k_a_ | **0.005** | 0.106 | 0.168 |  | k_a_ | **0.003** | 0.092 | 0.655 |
| k_v_ | **0.000** | **0.037** | 0.192 |  | k_v_ | **0.000** | **0.001** | **0.043** |
| k_p_ | **0.000** | 0.576 | 0.404 |  | k_p_ | **0.001** | **0.026** | 0.059 |
| k'_a_ | **0.001** | **0.014** | 0.107 |  | k'_a_ | **0.018** | **0.001** | 0.109 |
| k'_v_ | **0.003** | **0.000** | **0.043** |  | k'_v_ | **0.036** | **0.010** | 0.372 |
| k'_p_ | 0.237 | **0.046** | 0.667 |  | k'_p_ | 0.903 | 0.182 | 0.949 |
| k_s_ | **0.043** | 0.267 | **0.042** |  | k_s_ | 0.186 | 0.249 | 0.136 |

LG = lateral gastrocnemius; MG = medial gastrocnemius; SOL = soleus; TA = tibialis anterior

ka = acceleration gain; kv = velocity gain; kd = displacement gain; ka’ = prime acceleration gain; kv’ = prime velocity gain; kd’ = prime displacement gain; ks = stiction gain.

Significant differences are indicated in bold.

We are mainly interested in the fixed effect of group. To account for simultaneous interference, we performed a Bonferroni-Holm correction for the effect of group.

**Table P: Statistical outcome parameters (p-values, Fstat, and confidence intervals) for the fixed effect of group.**

| **Muscle** | **Parameter** | **p value** | **Fstat** | **CI** | | **Significant** | **p BH** | **BH - significant** |
| --- | --- | --- | --- | --- | --- | --- | --- | --- |
|  |  |  |  | **LB** | **UB** | **Y/N** |  | **Y/N** |
| LG | vP | **0.0027** | 9.4 | -0.804 | -0.173 | **Y** | 0.0710 | **Y** |
|  | v | **0.0035** | 8.8 | -0.915 | -0.183 | **Y** | 0.0083 | **Y** |
|  | p | **0.0184** | 5.7 | -2.937 | -0.275 | **Y** | 0.0100 | N |
|  | Pp | 0.1288 | 2.3 | -1.346 | 0.173 |  | 0.0125 | N |
|  | Ap | 0.2257 | 1.5 | -0.727 | 0.173 |  | 0.0167 | N |
|  | S | 0.4368 | 0.6 | -0.559 | 0.243 |  | 0.0250 | N |
|  | a | 0.5432 | 0.4 | -0.498 | 0.263 |  | 0.0500 | N |
|  |  |  |  |  |  |  |  |  |
| MG | vP | **0.0000** | 32.9 | -0.835 | -0.406 | **Y** | 0.0710 | **Y** |
|  | Ap | **0.0142** | 6.2 | -0.856 | -0.097 | **Y** | 0.0083 | N |
|  | v | **0.0371** | 4.4 | -0.754 | -0.024 | **Y** | 0.0100 | N |
|  | Pp | **0.0475** | 4.0 | -2.256 | -0.013 | **Y** | 0.0125 | N |
|  | a | 0.1056 | 2.7 | -0.095 | 0.979 |  | 0.0167 | N |
|  | S | 0.2669 | 1.2 | -0.804 | 0.224 |  | 0.0250 | N |
|  | p | 0.5746 | 0.3 | -1.807 | 1.006 |  | 0.0500 | N |
|  |  |  |  |  |  |  |  |  |
| SOL | v | **0.0005** | 12.9 | -0.810 | -0.234 | **Y** | 0.0710 | **Y** |
|  | Ap | **0.0012** | 10.9 | -0.946 | -0.238 | **Y** | 0.0083 | **Y** |
|  | vP | **0.0103** | 6.8 | -0.970 | -0.133 | **Y** | 0.0100 | N |
|  | p | **0.0262** | 5.1 | -2.469 | -0.185 | **Y** | 0.0125 | N |
|  | a | 0.0919 | 2.9 | -0.626 | 0.048 |  | 0.0167 | N |
|  | Pp | 0.1823 | 1.8 | -1.694 | 0.325 |  | 0.0250 | N |
|  | S | 0.2486 | 1.3 | -0.571 | 0.149 |  | 0.0500 | N |
|  |  |  |  |  |  |  |  |  |
| TA | vP | **0.0007** | 12.1 | -1.774 | -0.488 | **Y** | 0.0083 | **Y** |
|  | v | **0.0015** | 10.6 | -2.671 | -0.65 | **Y** | 0.0100 | **Y** |
|  | Pp | **0.0357** | 4.5 | -4.227 | -0.148 | **Y** | 0.0125 | N |
|  | a | 0.0986 | 2.8 | -1.577 | 0.136 |  | 0.0167 | N |
|  | p | 0.2588 | 1.3 | -2.794 | 0.758 |  | 0.0250 | N |
|  | Ap | 0.9376 | 0.0 | -0.283 | 0.262 |  | 0.0500 | N |

LG = lateral gastrocnemius; MG = medial gastrocnemius; SOL = soleus; TA = tibialis anterior.

BH = Bonferroni-Holm. Significant results are indicated with Y (yes) in column six before Bonferroni-Holm correction and in column nine after Bonferroni-Holm correction. New alpha-levels defined by the Bonferroni-Holm correction are indicated in column eight.


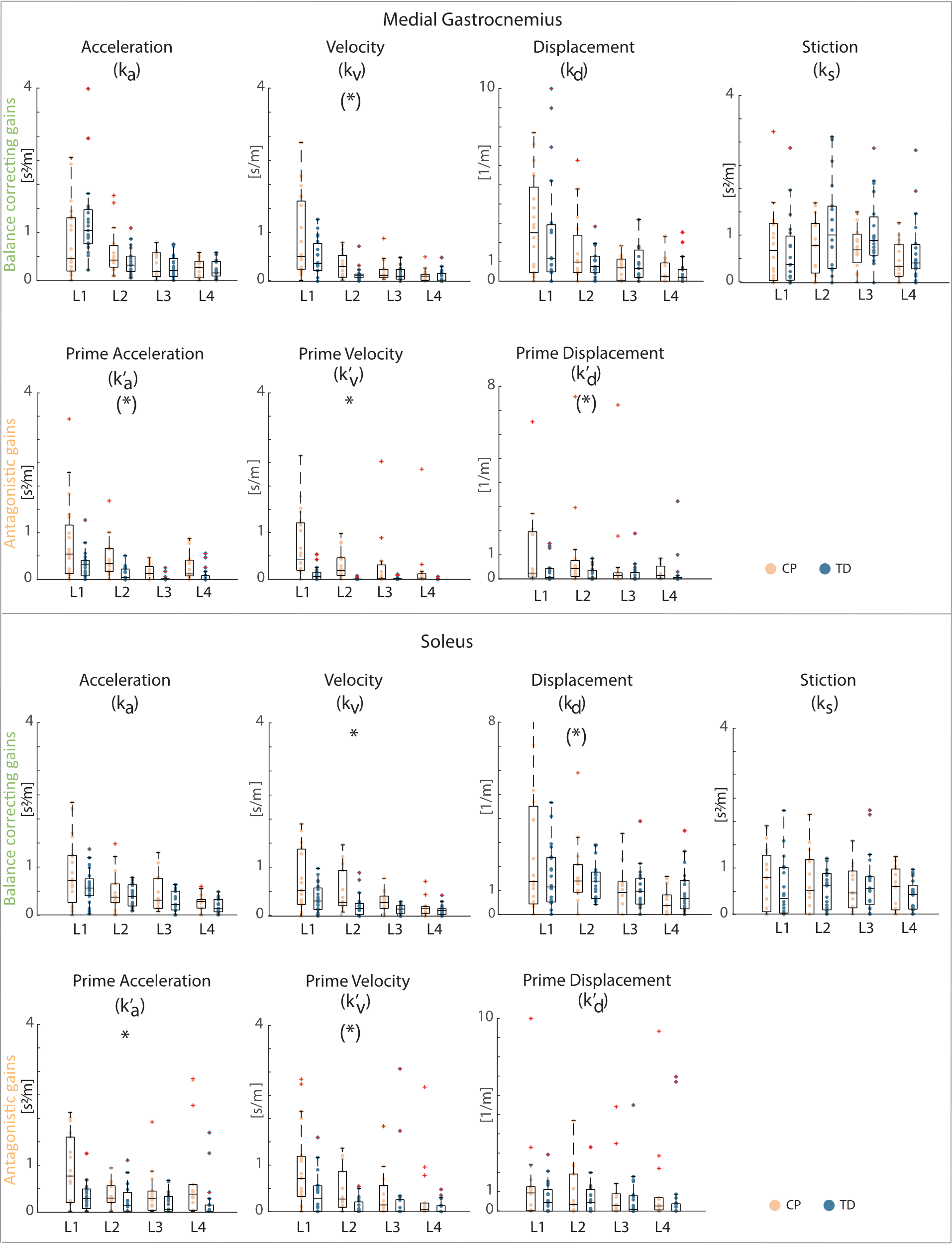

**Figure F: Center of mass feedback gains for all levels for children with cerebral palsy and typically developing children.** a) Medial gastrocnemius. b) Soleus.

Upper row: balance correcting pathway gains, bottom row: antagonistic pathway gains. L1-L4: level 1 to level 4. Boxplots in black indicate mean and interquartile ranges, and dots represent individual scores. Children with cerebral palsy (CP) in orange, typically developing (TD) children in blue. Significant differences between groups are indicated with a star after Bonferroni-Holm correction. A star between brackets indicates significant effects that did not survive the Bonferroni-Holm correction.

**S6. Correlation with MAS**

**
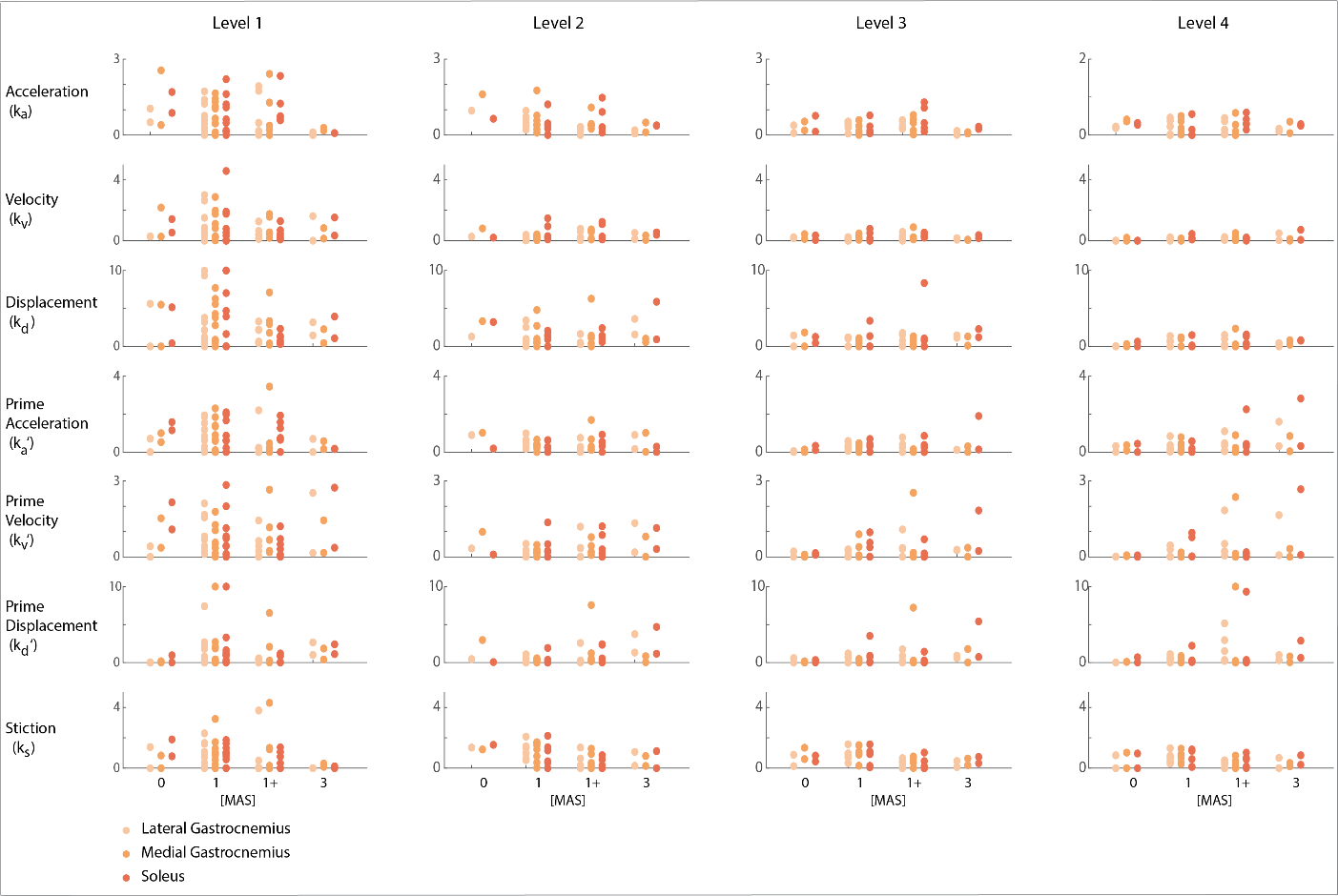
 Figure G: Associations between feedback gains for the lateral gastrocnemius, medial gastrocnemius and soleus and the Modified Ashworth Score of the gastrocnemii.** Dots are individual scores. Gains for lateral gastrocnemius in light orange, medial gastrocnemius in orange, soleus in dark orange.

*
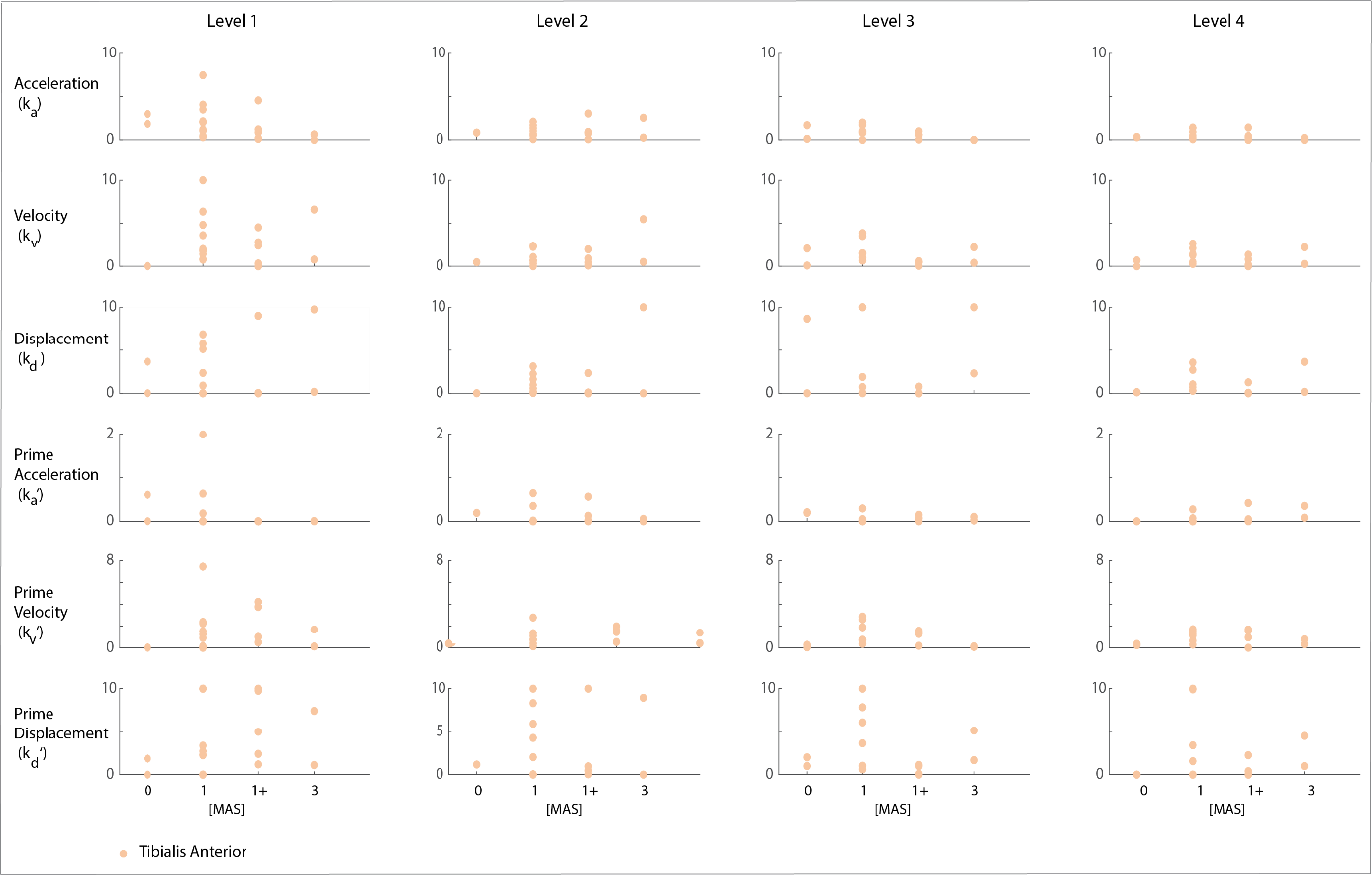
***Figure H: Associations between feedback gains for the tibialis anterior and the Modified Ashworth Score of the gastrocnemii.** Dots are individual scores.
