# Supplementary material for: Combined translational and rotational perturbations of standing balance reveal contributions of reduced reciprocal inhibition to balance impairments in children with cerebral palsy
Source: PLoS Comput Biol. 2024 Jun 13;20(6):e1012209. doi: 10.1371/journal.pcbi.1012209 (PMC11206838; doi:10.1371/journal.pcbi.1012209)
Supplement: Attachment — Submitted filename: Review1.pdf [file pcbi.1012209.s002.pdf]

This paper describes a study investigating responses to a combined rotational and translational perturbation to standing balance. The goal was to investigate if reciprocal inhibition is impaired in children with cerebral palsy, which can only be investigated by including rotation to the translational perturbation, since this causes a change in the centre of mass in backward instead of forward direction and therefore to separate the effect of reciprocal inhibition from an effective response with co-contraction.

There were some effects found that indeed the strategy used by CP children was due to a lack of reciprocal inhibition. However, the p-values were very close to 0.05. And since the same data was used for different tests, I wonder if these therefore actually provide a statistically significant difference. Furthermore, it would make sense to not only include the p-value, but also the power of the test to provide more confidence in these results.

The study is interesting, and the paper generally well written with nice figures. Some approaches in the analyses seem not well motivated to me, and some descriptions incorrect. Therefore, I would like to have these things clarified before publication. Despite the fact that I find the statistical evidence weak, I agree with the conclusion that these experiments provide evidence of reduced reciprocal inhibition. This conclusion is just not very well supported by the tests that were performed.

Major Comments

Figure 1: I did not initially see the light grey persons that show the expected different response for patients with CP. I would recommend a different, more obvious color.

Line 90-95: This explanation of reciprocal inhibition put me completely on the wrong foot. This text explain inhibition from the plantar flexors (SOL/GAS) to the dorsi flexors (TA), but the paper actually investigates the opposite inhibition (decreased activation in SOL/GAS due to TA activation, lines 505-506). Because of this explanation, I was looking for inhibition from plantar to dorsi flexors when analysing the paper, which was not actually the paper's topic. Therefore, I would strongly recommend aligning this explanation with the actual inhibition that is investigated. If my interpretation is incorrect, then please also clarify what the correct interpretation is.

Line 133-135: The first hypothesis includes two things (prolonged activity and increase co-activation). Since two different statistical tests are performed, it would make sense to split this hypothesis into two separate ones, each reflected by one statistical test.

Line 203-204: I did not necessarily see the increase/difference in the last two perturbations. In figure 2, the two darkest blue lines seem to overlap almost completely for platform translation and tilt rotation. Please highlight the difference between the two.

Line 206-207/374-375: was an investigation performed into if and how the lack of data on the CP side for large perturbations affected the different outcomes?

Line 211-218: Was any filtering applied to the marker data? If not, please specify.

Line 275-278: This mathematical equation does not have a unique solution. I think, based on line 300-301, that there should be an if-statement added to highlight that either pathway is active only when its output is positive. This is described in the text but should also be reflected in the mathematical equation.

Line 268: Was a sensorimotor response model created for each individual perturbation, or one for each child that participated?

Line 321: what is meant with Prime gains?

Line 342-343: please explain the difference between the simple and extended model.

Line 350-354: please motivate why this approach was used to test (I think) the first part of the first hypothesis: “that children with CP would have prolonged activity in the plantar flexors as well as increased muscle co-activation”. According to this paragraph, you test for differences in muscle activity among others, but a difference does not necessarily mean that activity was prolonged in specifically the plantar flexors.

Line 363: I disagree with the choice of not applying an adjustment for simultaneous inference. One cannot assume that tests are independent when they are taken on the same individuals performing the same motion. Therefore, some correction is required.

Line 366-369: which hypothesis is tested here?

Results: generally, the p-values are quite close to 0.05. Please provide additionally some information related to the power of the tests.

Minor Comments

Line 94-95: should one of the antagonistic be agonistic in this sentence? The sentence does not make sense to me now.

Line 114 + 117: the phrase “also investigate the response to rotational perturbations” in line 114, as well as the wording in line 117, imply that only rotational perturbations are going to be applied. However, the tests are applied to combined rotational and translational perturbations. Please update the wording to reflect what is actually being done.

Line 189-190: remove both (figure caption).

Line 205: I would recommend to add “the same” before “eight perturbations” to make this statement more specific.
