# Supplementary material for: Combined translational and rotational perturbations of standing balance reveal contributions of reduced reciprocal inhibition to balance impairments in children with cerebral palsy
Source: PLoS Comput Biol. 2024 Jun 13;20(6):e1012209. doi: 10.1371/journal.pcbi.1012209 (PMC11206838; doi:10.1371/journal.pcbi.1012209)
Supplement: Attachment — Submitted filename: 0.ResponseToReviewers_review1_v2.docx [file pcbi.1012209.s003.docx]

**Response to reviewers**

Thank you for this review.

Regarding the major comments of reviewer 1 and 2 about the statistics, we contacted L-Biostat, offering statistical consulting at KU Leuven, and discussed our statistical methods. They suggested to rerun our statistical analysis in order to account for unequal variances and to account for simultaneous interference across different statistical tests. Therefore, we performed new statistical analysis and added more statistical output parameters such as F-stats and confidence intervals. However, these new statistical tests did not change our conclusions. Please find detailed information below.

All specific issues are addressed below.

Indicated line numbers are in the simple markup style in word. Newly added text is underlined, while removed text is crossed out.

**Reviewer #1**

This paper describes a study investigating responses to a combined rotational and translational perturbation to standing balance. The goal was to investigate if reciprocal inhibition is impaired in children with cerebral palsy, which can only be investigated by including rotation to the translational perturbation, since this causes a change in the centre of mass in backward instead of forward direction and therefore to separate the effect of reciprocal inhibition from an effective response with co-contraction.

There were some effects found that indeed the strategy used by CP children was due to a lack of reciprocal inhibition. However, the p-values were very close to 0.05. And since the same data was used for different tests, I wonder if these therefore actually provide a statistically significant difference. Furthermore, it would make sense to not only include the p-value, but also the power of the test to provide more confidence in these results.

The study is interesting, and the paper generally well written with nice figures. Some approaches in the analyses seem not well motivated to me, and some descriptions incorrect. Therefore, I would like to have these things clarified before publication. Despite the fact that I find the statistical evidence weak, I agree with the conclusion that these experiments provide evidence of reduced reciprocal inhibition. This conclusion is just not very well supported by the tests that were performed.

Major Comments

Figure 1: I did not initially see the light grey persons that show the expected different response for patients with CP. I would recommend a different, more obvious color.

Thank you for this suggestion. We changed the color of the co-activation response to orange and indicated this in the figure description.

Line 90-95: This explanation of reciprocal inhibition put me completely on the wrong foot. This text explain inhibition from the plantar flexors (SOL/GAS) to the dorsi flexors (TA), but the paper actually investigates the opposite inhibition (decreased activation in SOL/GAS due to TA activation, lines 505- 506). Because of this explanation, I was looking for inhibition from plantar to dorsi flexors when analysing the paper, which was not actually the paper’s topic. Therefore, I would strongly recommend aligning this explanation with the actual inhibition that is investigated. If my interpretation is incorrect, then please also clarify what the correct interpretation is.

Thank you for this suggestion. In children with cerebral palsy, reciprocal inhibition is typically tested by applying a passive stretch to the plantar flexors (i.e., triceps surae) and investigate the response of the dorsiflexors (tibialis anterior). However, reciprocal inhibition also occurs during voluntary movement and happens in both directions between every pair of agonist-antagonists (i.e., inhibition of tibialis anterior upon plantar flexor activation and vice versa) (Yavuz 2018). With our sensorimotor model, we investigate reciprocal inhibition in both directions. Using the antagonistic pathways, we can estimate the lack of reciprocal inhibition in both muscles.

In the introduction, we described the most reported reciprocal inhibition mechanism. However, we understand the confusion and therefore we added some extra information on reciprocal inhibition in the other direction (from TA to plantar flexors) to this paragraph.

*L89-100:’Reduced reciprocal inhibition is often observed in CP [5-10] and could cause increased co-activation. Reciprocal inhibition is defined as the inhibition of antagonistic muscle activity upon agonistic muscle activation [11]. When the plantar flexors are activated by eliciting a stretch reflex, ~~in healthy adults~~ during rapid dorsiflexion of the ankle (i.e., stretching the plantar flexors by an examiner), ~~induces a strong response in the stretched muscle, while~~ the antagonistic muscle (i.e., tibialis anterior) remain silent in healthy adults whereas a response ~~In children with CP, such a strong response~~ is also observed in the antagonistic muscle with similar latencies as in the agonistic muscle in children with CP. This impaired reciprocal inhibition is also observed ~~both at rest and~~ with different levels of muscle activation [5,6]. When the tibialis muscle is activated to actively dorsiflex the ankle, the soleus muscle is inhibited in healthy adults [12], whereas there is no modulation of soleus inhibition during tibialis anterior contraction in children with CP. ~~A~~ Such reduction in reciprocal inhibition might also affect sensorimotor processing ~~in CP~~, (i.e., how the nervous system translates incoming sensory information about body motion into motor command to activate muscles) underlying reactive balance control in CP.’*

Line 133-135: The first hypothesis includes two things (prolonged activity and increase co-activation). Since two different statistical tests are performed, it would make sense to split this hypothesis into two separate ones, each reflected by one statistical test.

Thank you for this suggestion. We divided the first hypothesis (to assess whether co-activation is due to neural deficits and not a compensation strategy) in two parts:

1. We evaluated prolonged muscle activity of the plantar flexors. We expected that the plantar flexors would remain active upon reversal of the CoM movement in children with cerebral palsy but not in typically developing children. Here, we tested the interaction effect for average muscle activity between time bin and group.
2. We evaluated muscle co-activation. We expected that children with cerebral palsy would have increased muscle co-activation as compared to typically developing children. Here, we compared the co-contraction index between children with cerebral palsy and typically developing children.

*L140-145: ‘First – to assess whether co-activation is due to neural deficits and not a compensation strategy – we evaluated reactive muscle activity. We hypothesized that children with CP would have prolonged activity in the plantar flexors, i.e., we expected that the plantar flexors would remain active upon reversal of the CoM movement in children with CP but not in TD children. In addition we hypothesized that children with CP would have ~~as well as~~ increased muscle co-activation as compared to TD children.’*

Line 203-204: I did not necessarily see the increase/difference in the last two perturbations. In figure 2, the two darkest blue lines seem to overlap almost completely for platform translation and tilt rotation. Please highlight the difference between the two.

Thank you for this remark. The main difference between levels 3 and 4 is platform translational/rotational acceleration (and velocity). To make this more specific, we added an extra sentence to the figure description.

*L203-204: ‘Platform displacement increased from L2 to L3, while platform velocity and acceleration increased with every level.’*

Line 206-207/374-375: was an investigation performed into if and how the lack of data on the CP side for large perturbations affected the different outcomes?

Thank you for this question. We agree that we have a lot of missing data for children with cerebral palsy on the higher perturbation levels. It was difficult to decide how to handle these missing data, as the fact that the children were not able to perform the more difficult perturbations also includes information (i.e., their balance is probably more impaired compared to children with cerebral palsy that were able to perform the higher levels). We think that it is likely that children with more impaired control – and hence increased muscle co-activation - did not perform the higher levels. Therefore, we expect that if data was available for these children, it would strengthen our results. To address this issue, we added a small explanation in the discussion section.

*L599-603:*’Fewer children with CP than TD children were able to successfully perform the higher perturbation levels, leading to missing data in our analysis. It is likely that the children who were unable to perform the higher perturbation levels were more affected and therefore differed more from typically developing children. Given that we found differences between groups notwithstanding missing data from more affected children only strengthens our conclusions.‘

Line 211-218: Was any filtering applied to the marker data? If not, please specify.

Thank you for this question. Marker data was not filtered before applying the inverse kinematics tool in OpenSim. Within the OpenSim tool, we defined the weight of the markers based on their position (e.g., higher weight/trust on segment markers than anatomical markers) and their visibility. Joint angles were filtered (6Hz low pass filter) before calculating the center of mass position. Center of mass and ankle angle acceleration are calculated using the Savitzky-Golay filter (5^th^ order, with frame length 11). We added some details to this section to make it more specific.

*L226-231: ‘Joint angles were computed using OpenSim’s Inverse Kinematics tool. ~~And~~ CoM position was computed from low-pass filtered (6Hz) joint angles using OpenSim’s Body Kinematic tool. […] Ankle and CoM acceleration were then computed using a Savitzky-Golay filter (5^th^ order and frame length 11) [25].’*

Line 275-278: This mathematical equation does not have a unique solution. I think, based on line 300- 301, that there should be an if-statement added to highlight that either pathway is active only when its output is positive. This is described in the text but should also be reflected in the mathematical equation.

Thank you for this question. The half, open bracket is used to denote the positive part of the function (and thus replaces an if statement). We explain this on line 322: ‘Only the positive part of the signal ($\lfloor$.⌋) was used to represent excitatory drive to motor pools.’ This sign is used elsewhere (e.g., Welch and Ting 2009)

Line 268: Was a sensorimotor response model created for each individual perturbation, or one for each child that participated?

Thank you for this question. We created a sensorimotor response model (i.e., calculated the gains) for each participant and each perturbation level that was performed successfully. To make this more specific, we added an extra sentence to the method section. Evaluating subject- and task specific gains is in accordance with literature where they found subject-specific gains that differ with task difficulty (Welch & Ting 2008, McKay et al 2021).

*L349-350: ‘Gains were calculated for each participant and each perturbation level that was performed successfully.’*

Line 321: what is meant with Prime gains?

Thank you for this question. Prime gains are the gains calculated for the antagonistic pathways, while (‘normal’) gains are calculated for the balance correcting pathway. To make this more clear, we rewrote some sentences.

*Figure 4, figure description: ‘In the extended model, both pathways are used (~~gains and prime gains~~ balance correcting and antagonistic gains), while in the simple model only the balance correcting pathway is used.’*

*L344: ‘Gains were estimated by minimizing the cost function, which was defined as the weighted sum of the squared difference between reconstructed and measured EMG over a time interval from 0.5s before until 1.5s after perturbation onset (weight of 1) and squared prime gains (i.e., gains of the antagonistic pathway) (weight of 1E-4).*

*L345: ‘Prime gains (i.e., gains of the antagonistic pathway) were penalized to discourage the use of the antagonistic pathway unless this had a considerable effect on the fit between reconstructed and measured EMG as we did not expect the antagonistic pathway to be active in TD children.*

*L368-370: ‘To test this, we calculated the difference in cost (squared difference between reconstructed and measured activations) between the extended model with the antagonistic pathway (characterized by prime gains) and the simple model without antagonistic pathway (no prime gains). ~~Both models (with prime gains (extended model) and without prime gains (simple model)).~~*

Line 342-343: please explain the difference between the simple and extended model.

Thank you for this question. The simple model only consists of the balance correcting pathway (gains). This is the only pathway that is expected to contribute to muscle activity in typically developing children. In the extended model, we added an antagonistic pathway (prime gains) to account for antagonistic muscle activity, which is often observed in children with cerebral palsy but does not help to remain balanced. More concrete, the balance correcting pathway yields plantar flexor muscle activity when the center of mass moves forward and tibialis anterior activity when the center of mass moves backward. The antagonistic pathway yields plantar flexor muscle activity when the center of mass moves backward and tibialis anterior activity when the center of mass moves forward. To make this more concrete, we added an additional explanation to the method section.

*L290-293: ‘The balance correcting pathway yields plantar flexor activity when the CoM moves forward and tibialis anterior activity when the CoM moves backward. The antagonistic pathway yields plantar flexor activity when the CoM moves backward and tibialis anterior activity when the CoM moves forward.’*

*L367-370: ‘To test this, we calculated the difference in cost (squared difference between reconstructed and measured activations) between the extended model with the antagonistic pathway (i.e., prime gains) and the simple model without antagonistic pathway (i.e., no prime gains). ~~Both models (with prime gains (extended model) and without prime gains (simple model)).~~*

Line 350-354: please motivate why this approach was used to test (I think) the first part of the first hypothesis: “that children with CP would have prolonged activity in the plantar flexors as well as increased muscle co-activation”. According to this paragraph, you test for differences in muscle activity among others, but a difference does not necessarily mean that activity was prolonged in specifically the plantar flexors.

Thank you for this question. We tested whether children with cerebral palsy have prolonged activity in the plantar flexors by evaluating the interaction effect between time bin and group for the average muscle activity. A significant interaction effect indicates a difference between groups according to time bin. We found significant differences for this interaction effect, indicating higher muscle activity in the plantar flexors in time bin 3 for children with cerebral palsy compared to typically developing children. As typically developing children shut off their plantar flexor activity in time bin 3, we can interpret this difference as prolonged activity in the plantar flexors for children with cerebral palsy (continued plantar flexor activity in time bin 3). To make this more specific, we added an additional sentence to the Methods section.

*L379-381: ‘We investigated whether there was a significant interaction effect between group and time bin.’*

Line 363: I disagree with the choice of not applying an adjustment for simultaneous inference. One cannot assume that tests are independent when they are taken on the same individuals performing the same motion. Therefore, some correction is required.

Thank you for this remark. We agree that we stated this wrong initially. We contacted L-Biostat, offering statistical consulting at KU Leuven, and discussed the options for correcting for multiple testing. They suggested to perform Bonferroni-Holm corrections on the data. In a Bonferroni Holm correction, all p-values are ranked from lowest (most significant) to highest (not significant) values. The ‘new’ p-value for the lowest initial p-value is defined as the initial alpha ( = 0.05) divided by the number of tests (e.g. 7 for the SMR model of the LG). The ‘new’ p-value for the second lowest initial p-value is defined as the initial alpha divided by the number of tests -1. This process is continued until the higher p-value that is tested against 0.05. We performed the Bonferroni-Holm correction for each muscle separately as we expected different responses. A description of this multiple test correction is added to the methods section. Detailed statistical outputs including Bonferroni-Holm corrections were added to the supplementary material. Overall, conclusions were unaltered after adjusting for simultaneous inference.

*L400-401: ‘To account for simultaneous interference, we performed the Bonferroni-Holm correction.’*

Line 366-369: which hypothesis is tested here?

Thank you for this question. Here, we tested for the presence of antagonistic (i.e., balance disturbing) co-activation. If there is no antagonistic co-activation, the simple model and extended model would find the same solution, i.e. would equally well fit the data and thus have a similar cost. Antagonistic co-activation is not captured in the simple model. Therefore, if co-activation is present, the fit between modeled and experimental activation would be worse for the simple model compared to the extended model. We hypothesized that the decrease in the cost (i.e., improvements in the fit) would be larger for children with cerebral palsy compared to typically developing children, as children with cerebral palsy have more antagonistic co-activation. We states this more clear on L362-365.

*L362-365: ‘We also explicitly tested whether EMG trajectories could be reconstructed without using the antagonistic feedback pathways (i.e., activity of the plantar flexors when the CoM moves backward and activity of the tibialis anterior when the CoM moves forward) in both groups of children (Fig 4a). Antagonistic co-activation is not captured in the simple model. Therefore, if co-activation is present, the fit between modeled and experimental activation would be worse for the simple model compared to the extended model. We expected that the antagonistic pathway is not necessary to reconstruct reactive muscle activity in TD children, whereas adding the antagonistic pathway would improve the fit between reconstructed and measured EMG when reciprocal inhibition is impaired in children with CP.’*

Results: generally, the p-values are quite close to 0.05. Please provide additionally some information related to the power of the tests.

Thank you for this remark. An a priori power analysis was not possible because we could not estimate effect size based on literature. We added additional information to the statistical outcomes to allow for a better interpretation of the results. We added confidence intervals to the main text and figures when appropriate (otherwise to the supplementary material) and F-stats to the supplementary material. L-biostat (statistical consulting from KU Leuven) suggested to report F-stats as this comes closest to effect sizes. However, F-stats are hard to interpret and therefore, we opted to only present them in the supplementary material.

Minor Comments

Line 94-95: should one of the antagonistic be agonistic in this sentence? The sentence does not make sense to me now.

Thank you for this remark. Indeed, the second antagonistic need to be agonistic. I updated the sentence in the manuscript.

*L94:’In children with CP, such strong response is also observed in the antagonistic muscle with similar latencies as in the ~~antagonistic~~ agonistic muscle.’*

Line 114 + 117: the phrase “also investigate the response to rotational perturbations” in line 114, as well as the wording in line 117, imply that only rotational perturbations are going to be applied. However, the tests are applied to combined rotational and translational perturbations. Please update the wording to reflect what is actually being done.

Thank you for this remark. We rephrased the sentence on line 114. To investigate the use of co-activation, a rotational component, where co-activation is not useful, is needed.

*L117-120: ‘Yet to distinguish reduced reciprocal inhibition from the use of co-activation as a successful compensation strategy, we should also investigate the response to perturbations that contain a ~~the response to~~ rotational ~~perturbations~~ component.*

On line 124, we describe results from literature based on experiments that applied translational or rotational perturbations, without combining both. Therefore, we opted to not change our wording to reflect what is reported in literature.

We changed our wording in the discussion section on line 560.

*L551:‘Although increased muscle co-activation was observed in response to translational perturbations of standing balance in CP, this was not tested before during combined translational and rotational perturbations.’*

We also changed the wording in the author summary.

*L57-58: ‘Our combined translational and rotational perturbations induced a switch in center of mass movement (forward to backward).*

Line 189-190: remove both (figure caption).

Thank you for this suggestion. We deleted the word ‘both’ from the figure caption. In addition, we improved the quality of the figure.

*L202:’One perturbation involved simultaneous ~~both~~ translation and rotation of the platform.’*

Line 205: I would recommend to add “the same” before “eight perturbations” to make this statement more specific

Thank you for this suggestion. We followed your suggestion.

*L219: ‘Within each perturbation level, the same eight perturbations were administered with 12s between perturbations.’*

**Reviewer #2**

The proposed manuscript is a combined experimental and computational modeling study of the potential mechanisms of muscle co-contraction in children with cerebral palsy (CP). It uses translational and rotational perturbations to evaluate whether co-contraction of the TA and soleus are pathological consequences of CP or a motor control strategy. A computational model is then employed to test the hypothesis that co-contraction is caused by decreased reciprocal inhibition observed in children with CP. Th experimental design is quite clever and the use of modeling aids in overcoming some of the experimental limitations for examining mechanisms of CP symptoms. However, the study rationale is somewhat lost in the presentation of the introduction and there are some methodological choices that must be better justified. Finally, the statistical approach is incomplete and detracts from interpreting the data faithfully.

Major Issues

*Introduction*
- One of my primary concerns about the paper is that there is, in fact, two (admittedly related and interesting) studies here but they are being interwoven into a more elaborate story that makes the rationale of the study a bit convoluted. From what I can tell, there are two relevant observations from previous studies that motivate this one: 1) children with CP have increased muscle co-contraction and 2) children with CP have decreased reciprocal inhibition. The current study hypothesizes that 1) increased co-contraction is pathological, i.e. not a control strategy AND 2) that the increased co-contraction is caused by decreased reciprocal inhibition. The experimental paradigm tests the first and the modeling work tests the second. It is a reasonable rationale and approach, but it took me several reads to actually extract those couple sentences. (I actually just deleted an entire paragraph in this feedback critiquing the rationale of this study being fundamentally flawed because I misunderstood the rationale as it is currently presented.) I would recommend a rewrite that lays out the study rationale more linearly to avoid these confusions. I think it is critical to state that the experimental data tests only whether increased co-contraction is pathological vs a control strategy (there is no testing reciprocal inhibition at this point). It establishes that the co-contraction is not a control strategy and the subsequent modeling work implicates reciprocal inhibition.

Thank you for this suggestion. We updated the hypothesis section of the introduction in order to state this more clear.

*Introduction- L135-148: ‘Here, we combined translational and rotational perturbations to test whether muscle co-activation is the result of compensation or due to neural deficits. ~~Reduced reciprocal inhibition can explain alterations in balance control in children with CP.~~ If increased co-activation as observed in response to translational perturbations is not a compensation strategy, we expect to see it in response to combined translational and rotational perturbations as well. In that case, increased co-activation might be the result of ~~if~~ reduced reciprocal inhibition, ~~is reduced in children with CP, we expect to see increased,~~ which would be reflected in antagonists being activated along with agonists. ~~a co-activation and more specifically a reduced ability to suppress plantar flexor activity~~ ~~upon~~ ~~with activation of the tibialis anterior upon reversal of the CoM~~ ~~in response to combined backward translational and toe-up rotational perturbations.~~ First – to assess whether co-activation is due to neural deficits and not a compensation strategy – we evaluated reactive muscle activity. We hypothesize that children with CP would have prolonged activity in the plantar flexors, i.e., we expected that the plantar flexors would remain active upon reversal of the CoM movement in children with CP but not in TD children. In addition, we hypothesized that children with CP would have ~~as well as~~ increased muscle co-activation as compared to TD children. Second – to assess whether plantar flexor and tibialis anterior activity might have been driven by a common input due to reduced reciprocal inhibition- we evaluated the relation between CoM movement and reactive muscle activity using the sensorimotor response model described by Welch & Ting [13]. […]’*

*Methods*
- The sensorimotor response model should be better justified. Why is a relatively simple weighted sum of delayed COM acceleration, velocity, and displacement used to model EMG? What physiological, e.g. neuronal or musculoskeletal, properties or phenomenon motivate this model? Is the simple addition of a negative sign appropriate to create an ‘antagonist pathway’? Is it reasonable that COM be used to describe EMG for muscles which only span a limited number of joints?

Thank you for this remark. The sensorimotor model that we present here is based on extensive literature and in depth research by the group of prof. Lena H. Ting.

We rewrote this part in the introduction to make it more clear:

*L102-117: ‘~~We recently found that muscle activity in response to backward support surface translations during standing can be explained by delayed feedback of CoM kinematics in both TD children and children with CP, yet.~~ We found that both agonists and antagonists were more sensitive to CoM disturbances elicited by support-surface translations in children with CP than in TD children [4]. ~~Inspired by p~~ Previous work in healthy and pathological humans and animals [Welch & Ting 2009, Safavynia et al 2013, Payne et al 2021, McKay et al 2021] has shown that muscle activity in response to support-surface translations can be explained by CoM kinematics, with CoM displacement, velocity, and acceleration having a distinct effect on the muscle response. In a previous study, we reconstructed plantar flexor and tibialis anterior ~~reactive muscle~~ activity elicited by support-surface translations ~~of the plantar flexors~~ by a weighted linear combination of delayed (to account for neural transmission times) CoM displacement, velocity, and acceleration. ~~For the tibialis anterior,~~ We included ~~both a~~ stabilizing pathways that activate~~s~~ the plantar flexor and tibialis anterior ~~muscle~~ when the CoM moves forward and backward respectively, and a destabilizing pathway that activates the ~~muscle~~ tibialis anterior when the CoM moves forward (and plantar flexor activity is required to stabilize posture) [16]. The weights or gains of the CoM displacement, velocity, and acceleration in the linear combination indicate the sensitivity of the muscle response to CoM disturbances. We found that displacement and velocity gains were higher for plantar flexors and tibialis anterior in children with CP than in TD children [4]. The higher gains for the tibialis anterior for the destabilizing pathway reflect increased co-activation and might reflect reduced reciprocal inhibition, i.e., similar sensory input (CoM kinematics) drives both the agonist and antagonist.’*

In addition, Safavynia et al. showed that joint position, velocity, and/or acceleration cannot explain reactive muscle activity when perturbations elicit joint movements that are opposite to center of mass movements. This is described in the introduction L122-127:

*L122-127: ‘In healthy humans and animals, center of mass movement seems to dictate muscle responses to a wide range of translational and rotational support-surface perturbations. Translational and rotational perturbations that elicit similar center of mass movements yet different joint movements induce activity in similar groups of muscles [Ting & McPherson 2004, Diener 1983, Nardone 1990, Carpenter 1999]. Furthermore, delayed feedback from CoM kinematics but not joint angles could explain reactive muscle activity during a train of translational perturbations that induced uncorrelated CoM and joint angle trajectories [Safavynia 2013].*

To make it more specific in the hypothesis and methods section, we added the references there as well.

*L145-148:’ Second – to assess whether plantar flexor and tibialis anterior activity might have been driven by a common input due to reduced reciprocal inhibition- we evaluated the relation between CoM movement and reactive muscle activity, using the sensorimotor response model described by Welch & Ting [2009].*

*L288-290:’ We tested the relation between muscle activity and CoM kinematics by reconstructing measured EMG trajectories by a weighted sum of delayed CoM acceleration, velocity, and displacement trajectories [Welch and Ting 2009]. We modeled both a balance correcting and antagonistic feedback pathway (Fig 4a) [McKay et al 2021].*

- There is a severe overreliance on p-values in this paper and it results in a statistical approach that, while complex and extensive, undermines the results and the reader’s confidence in them. For starters, the statistical section does not seem to indicate the use of any corrections for multiple tests, which is very concerning. But even with these corrections, the huge number of inference tests being performed make it very difficult to be confident in each result. Ideally, I want to recommend that the authors remove the extraneous tests, focusing instead on the a priori hypotheses of the study and use more descriptive statistical metrics (confidence intervals, effect sizes, etc.) where appropriate. The purpose of hypothesis tests is just that – hypothesis testing.

Thank you for this remark. We contacted L-Biostat, offering statistical consulting at KU Leuven, and implemented the following changes to improve our statistical analysis.

First, we performed Bonferroni-Holm corrections to account for simultaneous interference. In a Bonferroni Holm correction, all p-values are ranked from lowest (most significant) to highest (not significant) values. The ‘new’ p-value for the lowest initial p-value is defined as the initial alpha ( = 0.05) divided by the number of tests (e.g. 7 for the SMR model of the LG). The ‘new’ p-value for the second lowest initial p-value is defined as the initial alpha divided by the number of tests -1. This process is continued until the higher p-value that is tested against 0.05. We performed the Bonferroni-Holm correction for each muscle separately as we expected different responses. A description of this multiple test correction is added to the methods section. Detailed statistical outputs including Bonferroni-Holm corrections were added to the supplementary material.

Second, we added additional information to the statistical outcomes to allow for better interpretation of the results. We added confidence intervals to the main text and figures when appropriate (otherwise to the supplementary material) and F-stats to the supplementary material. L-biostat suggested to report F-stats as these come closest to effect sizes. However, F-stats are hard to interpret and therefore, we opted to only present them in the supplementary material.

Finally, we focus only on the effects we are a priori interested in:

- Interaction effect between group and time bin for average muscle activity, center of mass kinematics, and ankle kinematics.

- Fixed group effects for muscle co-activation.

- Fixed group effects for parameters of the sensorimotor response model.

All additional fixed or interaction effects are reported in the supplementary material.

o One example of how this approach could be problematic is in Figure 6b. The manuscript states:

“Ankle angular velocity and acceleration were different between time bins and there was an interaction effect between time bin and group for most perturbation levels (velocity: L1, L2, L3, p < 0.02; acceleration: L1, L2, p < 0.05)….Visual inspection reveals a lower angular velocity and larger angular deceleration in the last time bin in children with CP”

This text states that there is a difference between time bins and then suggests that while there is no overall difference between the two groups, there is an interaction, i.e. the combination of group and time bin, which is significant. But we’ve already established that there are differences between time bins so having “some” of the interaction terms come up as significant really doesn’t indicate any type of difference between the groups. It is inappropriate to imply that “there are some differences between the groups in some time bins” based on the statistical testing used here. With this in mind, the statement “Visual inspection reveals a lower angular velocity and larger angular deceleration in the last time bin in children with CP” seems somewhat misleading. The decreased angular velocity is maybe a couple of degrees per second? Is that significant or clinically relevant, particularly with a limited sample number? Is the quality of the OpenSim InverseKinematics simulations so robust that this difference is above possible noise? It seems like if confidence intervals were used, they would likely be nearly identical. Can we really say these are different? I focused on Fig 6, but it applies to Fig 5 as well.

Thank you for this remark. We understand this point and rephrased this part.

*L435-442: ‘Ankle displacement, velocity, and acceleration was different between time bins but not between groups (Fig 6b, supplementary material S3.4 (S6 Table)). There was an interaction effect between time bins and group for angular velocity (L1-L3) (Supplementary material S3.4 (S7 – S8 Table). Angular velocity increased less from time bin 2 to 3 in children with CP than in TD children (Fig 6b). ~~Ankle angular velocity and acceleration were different between time bins and there was an interaction effect between time bin and group for most perturbation levels (velocity: L1, L2, L3, p < 0.02; acceleration: L1, L2, p < 0.05).~~While average ankle angular displacement and velocity continued to increase from the second to the third time bin, average ankle angular acceleration decreased from the second to third time bin (Fig 6b). Hence, ankle angular acceleration is another potential trigger for the switch between plantar flexor and TA activity.’*

Forgive me for pontificating on this point so fervently, but I feel it is important. I would suggest that the authors revisit their statistical tests and report not only the p-value but the effect sizes and confidence intervals as well and use the entirety of information to inform their conclusions.

*Results*
- In Figure 5 and 6, it looks as though average values are displayed for each time bin, but these are actively modulating signals (EMG and kinematics). Is it appropriate to simply average across this whole time period?

Thank you for this remark. We agree that the average value does not capture all the information in the signal. We chose this summary value because it is easy to interpret in terms of how much the muscle is on. The sensorimotor response model is based on time trajectories (relating kinematics and muscle activity) and in that sense complements the average values.

*Discussion*
- I think it would be worthwhile for the authors to elaborate on the implications of their findings, particularly for treatment of CP. Any speculation should be explicitly stated as such, but it would be good to provide a little more context to the paper’s findings.

Thank you for this suggestion. We added a small paragraph to the discussion section to elaborate on the clinical implications of our results.

*Discussion L604-612: ‘Our results might have clinical importance and give inspiration to therapists to explore new training options. Currently, clinical assessments mainly focus on hyper-reflexia whereas reduced reciprocal inhibition is less evaluated. Based on our results, we suggest that clinical assessments of reduced reciprocal inhibition might provide useful information about balance control impairments. Therapists should be aware of the underlying mechanisms of muscle co-activation during balance control in order to develop novel therapeutic approaches. These new therapies should specifically target reciprocal inhibition as our results suggest that improved reciprocal inhibition might improve balance control in children with CP. ‘*

Minor Issues

- How are the time bins (Z1, Z2, and Z3) selected? Based on physiology?

Thank you for this question. The different time bins are based on literature. Gollhoffer et al. (1986) described physiological reactions during combined translational and rotational perturbations. We adapted these time bins to our perturbation profiles based on the observed physiological reactions across the typically developing children.

Z1: Only little reactive muscle activity due to the slow movement onset of the platform and neural transmission delay.

Z2: First peak in plantar flexor activity in typically developing children.

Z3: Switch between plantar flexor and tibialis anterior activity in typically developing children.

To make this more specific, we added an extra sentence.

*L246-248: ‘Time bins were defined based on the sequence of muscle responses during combined translational and rotational perturbations described in literature for healthy participants [16,23].’*

- Figure 3 & 4: While I appreciate the authors’ transparency in labelling their example as ‘exemplar’ but it immediately begs the question, “So if these are the ‘best’, what do the typical examples look like?” I admit there are already a lot of plots in these figures so maybe including other examples in the supplementary would be useful. One approach is to include three examples: best, representative, and worst.

Thank you for this suggestion. The figures demonstrated representative examples, and not ‘the best’ responses. However, we agree with your suggestion. Based on the co-contraction index during perturbation level 2 (see figure below), we selected 2 children with low co-activation (light blue dots in figure below), two children with average co-activation (yellow dots in figure below), and two children with high co-activation (red dots in figure below). Figure 3 and 4 in the manuscript represent the children with average co-activation. In supplement, we added data from 2 children with high co-activation (S3 Fig, S5 Fig) and two children with low co-activation (S2 Fig, S5 Fig).

Figure: Co-contraction index for level 2 of combined translational and rotational perturbations. Exemplar subjects used for figures in the manuscript and supplement are indicated with blue dots (low co-activation), yellow dots (average co-activation), and red dots (high co-activation).

- Figure 3: I’m a bit confused what the bar plots actually show? Are they the mean of each time bin for the corresponding traces to the left? It would be good to include this information in the legend.

Thank you for this question. Indeed, the bars represent the mean for each time bin for the corresponding average (black) trace on the left. To make this more specific, we added an extra sentence to the figure description.

*L274-275: ‘The bars represent the average of each time bin for the corresponding (black) trace (average over trials) on the left.’*
