# Supplementary material for: Combined translational and rotational perturbations of standing balance reveal contributions of reduced reciprocal inhibition to balance impairments in children with cerebral palsy
Source: PLoS Comput Biol. 2024 Jun 13;20(6):e1012209. doi: 10.1371/journal.pcbi.1012209 (PMC11206838; doi:10.1371/journal.pcbi.1012209)
Supplement: Attachment — Submitted filename: 0.ResponseToReviewers_review2_v2.docx [file pcbi.1012209.s004.docx]

**Response to reviewers**

Thank you for this review.

All specific issues are addressed below. Newly added text is underlined, while removed text is crossed out.

Line numbers refer to the Track-changes manuscript.

**Reviewer #1**

Overall, the previous comments have been addressed well, and I like the paper and its contribution. I just have two general comments where I think some extra explanation is required, and some smaller comments about writing/grammar.

General Comments

Line 425-426: Is the EMG normalization affected by the fact that not all patients were able to complete the protocol? If the perturbations are not as large for some participants as for others, while the maximum across all is used for normalization, it seems possible to me that this normalization could affect the results. Has this been investigated?

Thank you for this remark. We acknowledge the inherent difficulty of consistently scaling EMG across different subjects. Our decision to scale to the maximum across all perturbation levels is based on the underlying assumption that there might be big differences in EMG magnitude for a given perturbation level between TD children and children with CP whereas differences might be smaller closer to their maximal performance (i.e. at the maximal perturbation level they could withstand without stepping). Typically developing children and some less impaired children with CP could perform all perturbations without stepping and might have not have reached their maximum activation. This would result in lower scaling factors and higher EMG values for TD children and less impaired children with CP and therefore, any errors introduced by this method would lead to an underestimation of the observed differences between groups.

In a related study investigating the co-contraction index during different perturbations, we evaluated the effect of different scaling factors (e.g., maximum across all perturbation levels, maximum across all perturbation levels and functional movements, lowest level of the perturbations, highest perturbation level). We did not find any differences in ranking across subjects (i.e., child with high co-activation still had high co-activation independent of different scaling factors). Taken together, we assume that the scaling method did not affect our overall conclusion.

To make this more clear, we added a short explanation in the discussion section:

L612:’ […] The maximum EMG across all perturbations levels was used for scaling and therefore missing data might have impacted EMG scaling. However, it is unlikely that EMG magnitude at a given perturbation level is similar across children given the differences in muscle responses that we observed. Therefore, our scaling method is based on the assumption that the EMG magnitude is similar at maximal performance, i.e., at the highest perturbation level that could be performed without stepping. Many TD children might have been able to withstand perturbations larger than those admitted here without stepping. Hence, we might have used scaling factors that are too low, resulting in an overestimation of the muscle responses and thus an underestimation of the differences between groups.’

Line 580-581: here, only the COM is written as a potential trigger, while the results have shown that also the ankle angular acceleration is a potential trigger. Please motivate why only the COM is listed in the discussion.

Thank you for this question.

Our results indicate that ankle acceleration potentially have a large contribution to CoM acceleration. However, literature shows that joint angles (velocity and acceleration) alone cannot explain muscle activity in response to standing balance perturbations in healthy adults, whereas CoM kinematics can explain reactive muscle activity.

To explain this further, we added a short paragraph to the discussion section:

L551-555: ‘We did not explore models based on feedback from joint instead of CoM kinematics because previous work has demonstrated that models based on feedback from joint kinematics could not explain muscle responses across perturbation conditions [Safavynia et al. 2013], However, CoM and ankle kinematics might be related in some parts of the response (see e.g., in Fig. 6) and hence the switch from plantar flexor to dorsiflexor activity also coincides with a change in sign of ankle angular acceleration.

Writing Comments

Line 93-98: this sentence is complicated. I would suggest splitting it into two.

Thank you for this remark. We changed this part.

L91-96: ‘In healthy adults, ~~When the plantar flexors are activated by eliciting a stretch reflex, during~~ rapid dorsiflexion of the ankle elicits reflex activation of the ~~(i.e., stretching the~~ plantar flexors (stretched muscles) ~~by an examiner)~~ while the antagonistic muscle (i.e., tibialis anterior) remains silent. In contrast, in children with CP, ~~in healthy adults whereas,~~ rapid dorsiflexion of the ankle elicits a response ~~is also observed in the antagonistic muscle~~ with similar latencies in both plantar flexors and tibialis anterior.

Line 142-148: consider rephrasing as the sentence is quite complex.

Thank you for this suggestion. We tried to make the sentence more easy to read.

L136: ‘Here, we combined translational and rotational perturbations to test whether muscle co-activation is the results of compensation or due to neural deficits. If increased co-activation ~~as observed~~ in response to translational perturbations ~~is not a compensation strategy~~ is due to neural deficits, we expect to see it in response to combined translational and rotational perturbations as well. In that case, the increased co-activation might be the results of reduced reciprocal inhibition leading to simultaneous activation of agonists and ~~, which would be reflected in~~ antagonists ~~being activated along with agonists~~. ’

Line 172: Please rename section to Participants

We changed the title of this section.

Line 208: connected instead of adjusted?

Thank you for this suggestion. We changed this.

L200: ‘Children were secured using a safety harness, ~~adjusted~~ connected to an overhead rail to prevent falling.

Figure 2 caption, 2nd line: exemplar reactive balance responses

Thank you for this suggestion. We changed it.

L203: ‘[…] b) platform kinematics and exemplar reactive balance ~~exemplar~~ responses […]’

Line 247-248: was this maximum value before or after filtering?

Thank you for this question. We used the maximum value observed across all perturbations after filtering.

L239: ‘The filtered EMG was scaled by the maximum, filtered value across all perturbations for every participant.’

Line 333-334: I would put this information closer to the equation as it is quite relevant for the equation.

Thank you for this suggestion. We moved this sentence to line 316-317.

Line 416-419: I did not understand this part. Consider rephrasing.

Thank you for this remark. We rephrased this part to make it more clear.

L400: ’To account for unequal variances, we incorporated weights w ~~in the model~~ for each observation (i) that is used as input to the model ~~(e.g., parameter kA for TD number and level x, EMG for CP number n and time bin x)~~. Weights ~~are~~ were calculated as the inverse of the variance observed across all observation within one group (i.e., CP or TD) and condition (i.e., different time zones or perturbation level) (yi): w = 1/var(y).

Line 451 and 459: were instead of was

Thank you for this remark. We changed it.

L431: ‘CoM displacement, velocity, and acceleration ~~was~~ were different between time bins but not between groups, […]’

L439:’Ankle displacement, velocity, and acceleration ~~was~~ were different between groups […]’

Figure 7: what does the light grey mean in the figure?

As mentioned in the figure description, grey bars indicate group averages. However, we acknowledge that it might be confusing to both show median and average values. Therefore, we opted to delete the group averages (grey bars) from the figure. This way the figure is also more consistent with other figures in the manuscript.

Line 649: evaluated less (word order)

Thank you for this suggestion, we changed it.

L623:’[…] whereas reduced reciprocal inhibition is ~~less~~ evaluated less.’

**Reviewer #2**

The authors have appropriately addressed my previous feedback and concerns. The introduction more clearly articulates the study rationale and hypotheses as well as the previous work that formulated the study. Furthermore, I applaud the changes to the statistical approach which is much more comprehensive and, in my opinion, adds greater confidence to the findings.

Thank you.
